# Supplementary material for: Oriented arrangement of simple monomers enabled by confinement: towards living supramolecular polymerization
Source: Nat Commun. 2021 May 10;12:2596. doi: 10.1038/s41467-021-22827-4 (PMC8110532; doi:10.1038/s41467-021-22827-4)
Supplement: Supplementary file 1 — Supplementary Information [file 41467_2021_22827_MOESM1_ESM.pdf]

Supplementary Information

Oriented Arrangement of Simple Monomers Enabled by Confinement: Towards Living Supramolecular Polymerization

Yingtong Zong[†], Si-Min Xu[†], Wenying Shi*[, †] and Chao Lu*[, †]

[†]State Key Laboratory of Chemical Resource Engineering, Beijing University of Chemical Technology, 15 Beisanhuan East Road, P. Box 98, 100029, Beijing (P. R. China).

*E-mail: shiwy@mail.buct.edu.cn; luchao@mail.buct.edu.cn

CONTENTS

1. Supplementary Methods.. S7

Chemicals and Materials S7

Apparatus S7

Preparation of LDHs with different sizes S8

Preparation of SG7-LDH S8

Preparation of LSP S9

Self-replication of LSP_{20} S9

The effect of dilution on the formation of LSP_{20} S9

Reversibility of LSP S10

Control experiments S10

Tuning the size of LSP via LDH confinement S10

Tuning the size of LSP via solvent processing S10

Tuning the size of LSP via mechanical agitation S11

Preparation of SSP S11

Regulation of the size of SSP_{20} S11

Living nature of seed-induced polymerization based on metastable LSP_{20} S12

Chiral recognition of metastable LSM_{3000} to L- or D-Arg S12

Universality experiments S13

Sample preparation S13

Model construction S14

Computational methods S15

2. Supplementary Figures S17

Figure S1. UV spectra of SG7-LDH and SG7 solution S17

Figure S2. FL emission spectra of SG7 solution S18

Figure S3. XRD patterns of Cl-LDH precursor and SG7-LDH S19

Figure S4. Polarized FL profiles and anisotropic value (*r*) for SG7-LDH S20

Figure S5. Polarized FL profiles and anisotropic value (*r*) for SG7 powder S21

Figure S6. The snapshot of SG7-LDH after AIMD simulations of 100 ps S22

Figure S7. FL emission spectra of SG7-LDH S23

Figure S8. FL emission spectra of metastable LSM_{20}–LSM_{3000} S24

Figure S9. FL emission spectra of metastable LSM_{20} in Condition (3) and (4) S25

Figure S10. Temperature-dependent absorption of metastable LSP_{20} S26

Figure S11. FL emission spectra of elongated LSP_{20} S27

Figure S12. FITS of freshly sonicated LSP S28

Figure S13. Schematic presentation of experimental set-up of FITS S29

Figure S14. FL excitation spectra of LSP_{20}–LSP_{3000} S30

Figure S15. SEM images of LSP_{20} with ultrasound elongated for different time S31

Figure S16. FL spectra of fresh SSP_{20} made from LSP_{seed} with different concentrations S32

Figure S17. FL emission spectrum of SG7 solution S33

Figure S18. FL emission spectrum of SG7 monomer and $SG7_{agg}$ S34

Figure S19. SEM images of metastable LSP_{20} and SSP_{20} S35

Figure S20. FL spectra of the reversible disassembly and re-assembly of LSP_{20} S36

Figure S21. FL spectra of SG7 powder dispersed in CH_3OH/TFA (1:2.5 v/v) S37

Figure S22. Time course of emission wavelength of SG7 solution.. S38

Figure S23. FL spectra of diluted LSM in CH_3OH/TFA (1:2.5 v/v)...................................... S39

Figure S24. FL spectra of transformation from LSM to LSP in CH_3OH/TFA (1:2.5 v/v)....... S40

Figure S25. XRD patterns of SSP, LSP, LSM and control samples.. S41

Figure S26. Polarized FL profiles and anisotropic value (*r*) for SG7-LDH_{20}-surface............. S42

Figure S27. SEM images of SP_{20} made from SG7-LDH_{20}-surface S43

Figure S28. FL spectra of SP_{20}... S44

Figure S29. SEM images of control samples.. S45

Figure S30. TEM images of pure LDH precursors.. S46

Figure S31. SEM images of pure LDH precursors and intercalated LDHs............................. S47

Figure S32. SEM images of SG7 LSM_{20} in equilibrium state in CH_3OH/TFA (5:3 v/v)......... S48

Figure S33. Polarized FL profiles and anisotropic value of LSP_{20}−LSP_{3000} S49

Figure S34. SEM images of metastable LSP_{20}−LSP_{3000} in batch 1−3.................................... S50

Figure S35. FL spectra of transformation from LSM_{20}−LSM_{3000} to LSP_{20}−LSP_{3000} S51

Figure S36. Schematic illustration of pathway of metastable LSP_{20}−LSP_{3000}.......................... S52

Figure S37. Time-dependent FL spectra of LSP_{20} in Condition (2) S53

Figure S38. SEM images of products in Condition (1)−(4)... S54

Figure S39. SEM images of LSP_{20} without ultrasound S55

Figure S40. CLSM images of metastable LSP_{20} without ultrasound....................................... S56

Figure S41. SEM images of LSP_{20} elongated for 12 h with different ultrasound time S57

Figure S42−44. SEM images of fresh SSP_{20} controlled by concentration................................ S58

Figure S45. SEM images of fresh SSP_{50}−SSP_{3000} S61

Figure S46. Optimized geometries of ordered SG7 in LDH S62

Figure S47. UV-vis spectra of LSP_{20}−LSP_{3000} S64

Figure S48−49. Absorption spectra of metastable LSP_{20} S65

Figure S50−53. FTIR spectra of SG7 powder, SG7-LDH and LSP S68

Figure S54. Photos of LSP and SG7 powder dispersed in different solvent S73

Figure S55−56. FL emission spectra of chiral recognition products by LSM_{3000} S74

Figure S57. CLSM images of chiral recognition products by LSM_{3000} S76

Figure S58. Optimized geometries of LSM_{3000}+L and LSM_{3000}+D S77

Figure S59. FL excitation spectra of chiral recognition products in three batches S78

Figure S60−61. FL emission spectra of chiral recognition products by LSM_{100} S79

Figure S62. FL spectra of chiral recognition products by LSM_{20} or LSM_{50} S82

Figure S63. XRD patterns of other intercalated LDHs S83

Figure S64−66. UV-vis spectra of other intercalated LDHs S84

Figure S67−69. Polarized FL profiles and anisotropic value for other intercalated LDHs S87

Figure S70−74. The AIMD simulations results of other intercalated LDHs S90

Figure S75. Schematic illustration for the definition of θ S95

Figure S76. The top view of all intercalated LDHs S96

Figure S77. The side view of all intercalated LDHs S97

Figure S78. Schematic illustration for university section S98

Figure S79−83 SEM images of LSP and contrast samples in university section S99

3. Supplementary Tables S104

Table S1. ICP-MS results of LDH precursors S104

Table S2. Elemental analysis results of SG7-LDH and SG7-LDH_{20}-surface S105

Table S3. A_w, A_n and PDI of metastable LSP_{20}−LSP_{3000} S106

Table S4−5. Parameters in Debye plot S107

Table S6. Comparison of size or DP between previous work and our work S109

Table S7. The elemental analysis results of all intercalated LDHs S110

4. Supplementary References S111

1. Supplementary Methods

Chemicals and Materials. $MgCl_2 \cdot 6H_2O$, $AlCl_3 \cdot 6H_2O$, NaCl, NaOH, CH_3OH, CH_3CN and urea were purchased from Beijing Chemical Reagent Company (Beijing, China). Trifluoroacetic acid (TFA), 8-hydroxypyrene-1,3,6-trisulfonate (solvent green 7, SG7), D-arginine (D-Arg), L-arginine (L-Arg), benzenesulfonic acid (BSA), 3-aminobenzene sulfonic acid (3-ABSA), 2,5-diaminobenzenesulfonic acid (2,5-DABSA), Congo red (CR) and 6,7-dihydroxynaphthalene-2-sulfonate (DHNS) were obtained from J&K Chemical (Beijing, China). In this work, all reagents were used as received without further purification. All solutions were prepared with deionized water.

Apparatus. The powder XRD measurements were carried out on XRD-6000 (Shimadzu, Japan) with a scan rate of 10º min^{-1} and a scan scope ranging from 5º to 90º for 2θ angle. FTIR spectra were measured on a Nicolet 6700 FTIR spectrometer (Thermo, America). SEM images were characterized by HT7800 (Hitachi, Japan). TEM images were obtained from HT7700 (Hitachi, Japan) and HRTEM images were obtained from JEM-ARM200F (JOEL, Japan). SLS results were tested on a Zetasizer 3000HS nanogranularity analyzer (Malvern Instruments, UK). UV-vis spectra were recorded on U-H3900 (Hitachi, Japan). Temperature-dependent absorption spectra were performed on a J-815 spectrophotometer (Jasco, Japan). FL spectra were recorded on FS5 (Hitachi, Japan). FITS results were tested by F-7000 (Hitachi, Japan). Polarized FL profiles and anisotropic value (r) were tested on FLS980 (Edinburgh, UK). CLSM images were obtained by a Leica TCS SP8 confocal laser scanning microscope (Leica, Germany). ICP-MS results were obtained by iCAP6300 Radial (Thermo Fisher Scientific, US). The elemental analysis was tested on vairo EL CUBE (Elementar, Germany).

Preparation of LDHs with different sizes. Mg_3Al-Cl-LDHs (~20 nm) were prepared *via* the co-precipitation method. Briefly, NaCl solution (1.00 M) of 50.0 mL was firstly added into a 500 mL four-necked flask. 50.0 mL of salt solution containing 3.75 mmol $MgCl_2 \cdot 6H_2O$ and 1.25 mmol $AlCl_3 \cdot 6H_2O$ was marked as solution A. 50.0 mL NaOH solution (1.00 M) was marked as solution B. Both of them were added dropwise into the flask under vigorous stirring simultaneously. In the whole process, the pH value of the suspension remained at ~8.00. The mixture was kept stirring and aged under N_2 atmosphere for 24 h at room temperature. Mg_3Al-Cl-LDHs (~50 nm) were prepared by the following method. NaOH solution (0.500 M) was dropped into salt solution (50.0 mL) containing 30.0 mmol $MgCl_2 \cdot 6H_2O$ and 10.0 mmol $AlCl_3 \cdot 6H_2O$ until pH ≈ 8.50. And then, the mixture was transferred into a Teflon-lined stainless steel autoclave, heated at 110 °C for 24 h. Mg_3Al-Cl-LDHs (~100 nm) were prepared *via* the same method as 20 nm LDHs except that all the concentrations of salt solutions were increased by 10 times. The concentration of NaOH solution was appropriately increased to 3.00 M. The Mg_3Al-CO_3-LDHs (~3 μm) were synthesized in the presence of urea. The salt solution (70.0 mL) containing 4.00 mmol $MgCl_2 \cdot 6H_2O$, 1.00 mmol $AlCl_3 \cdot 6H_2O$ and 20.0 mmol urea was added into Teflon-lined stainless steel autoclave, heated at 100 °C for 24 h. Mg_3Al-Cl-LDHs (~3 μm) were obtained by ion-exchange of Mg_3Al-CO_3-LDHs (0.300 g) in NaCl solution (1.00 M, 300 mL, pH = 6.50) under N_2 atmosphere for 48 h. Finally, all Mg_3Al-Cl-LDHs mentioned above were washed with deionized water for three times and dried in vacuum drying oven at 70 ºC. All water used in the process was treated with distillation.

Preparation of SG7-LDH. 0.313 g Mg_3Al-Cl-LDH with different sizes were added into SG7 solution (12.5 mM, pH = 8.00) at 80 ºC under N_2 atmosphere, respectively. SG7-LDHs were

obtained by ion-exchange for 24 h. 0.313 g Mg_3Al-CO_3-LDH (20 nm) were added into SG7 solution (12.5 mM, pH = 8.00) at 25 ºC under N_2 atmosphere. SG7-LDH_{20}-surface was obtained by ion-exchanging for 24 h. The products were washed with deionized water and CH_3OH respectively for three times and re-dispersed in CH_3OH as stocking solution (20.0 g L^{-1}) for further use ([SG7] = 10 mM). All water used in the process was treated with distillation.

Preparation of LSP. The ordered SG7 in the confinement of LDHs could be released from as-prepared SG7-LDH (4 mg) in 320 μL mixture solvent CH_3OH/TFA (5:3 v/v), which is defined as the metastable LSM. The metastable LSP was obtained by further increasing the amount of TFA until CH_3OH/TFA (1:3 v/v), followed with ultrasound for 1 h to expose more active sites (The control experiment is metastable LSP without ultrasound). After standing for 12 h, the corresponding energetically favored states of LSP were achieved. During ultrasound, the mixture of ice and water was used to keep a constant temperature at 0 ºC.

Self-replication of LSP_{20}. The as-prepared SG7-LDH_{20} with different amounts was dissolved in CH_3OH/TFA (1:3 v/v) to prepare metastable LSP_{20} under low concentration ([SG7] = 0.15 mM~0.25 mM). The nonlinear sigmoidal increase (self-replication characteristic) of them can be obtained by temperature-dependent absorption during their assembly.

The effect of dilution on the formation of LSP_{20}. In addition, the dilution to metastable LSM had no effect on the transformation from metastable LSM to metastable LSP. Firstly, the metastable LSM ([SG7] = 2.86 mM) was prepared by dissolving 4 mg SG7-LDH_{20} into 700 μL CH_3OH/TFA (1:2.5 v/v). The metastable LSM ([SG7] = 1.43 mM) can be obtained by adding equal volume of CH_3OH/TFA (1:2.5 v/v) to dilute metastable LSM ([SG7] = 2.86 mM), so did metastable LSM ([SG7] = 0.715 mM). After dilution, the transformation from metastable LSM

([SG7] = 0.715 mM) to metastable LSP was achieved by adding more TFA to CH_3OH/TFA (1:3 v/v).

Reversibility of LSP. The metastable LSP_{20} was firstly prepared by dissolving 4 mg SG7-LDH_{20} into 800 μL CH_3OH/TFA (1:3 v/v). Firstly, the disassembly of metastable LSP_{20} happened after adding 100 μL good solvent (CH_3OH) into 800 μL CH_3OH/TFA (1:3 v/v) to achieve CH_3OH/TFA (1:2 v/v). After then, the disassembled metastable LSP_{20} could re-assemble after adding 300 μL poor solvent (TFA) into 900 μL CH_3OH/TFA (1:2 v/v) to achieve CH_3OH/TFA (1:3 v/v).

Control experiments. The inactive SP_{20} and active mixed SP_{20} are prepared by the same method of above LSP. The inactive SP_{20} is made from SG7-LDH_{20}-surface. The adsorbed SG7 could be released from SG7-LDH_{20}-surface (4 mg) in 320 μL mixture solvent CH_3OH/TFA (5:3 v/v). The SP_{20} was obtained by further increasing the amount of TFA until CH_3OH/TFA (1:3 v/v), followed with ultrasound for 1 h. To obtain active mixed SP_{20}, SG7-LDH_{20}-surface (2 mg) and SG7-LDH_{20} (2 mg) were physically mixed in 800 μL mixture solvent CH_3OH/TFA (1:3 v/v), followed with ultrasound for 1 h. During ultrasound, the mixture of ice and water was used to keep a constant temperature at 0 ºC.

Tuning the size of LSP *via* LDH confinement. The metastable LSPs are prepared from LDHs (4 mg) with different size (20, 50, 100 and 3000 nm) in 800 μL mixture solvent CH_3OH/TFA (1:3 v/v), followed with ultrasound for 1 h, which are defined as the metastable LSP_{20}, LSP_{50}, LSP_{100} and LSP_{3000}.

Tuning the size of LSP *via* solvent processing. The different kinetic assemblies of metastable LSPs were obtained by change timing of addition of the bad solvent (TFA) into

SG7-LDH (4 mg in 200 μL CH_3OH) during self-assembly. Condition (1) 0 min with 600 μL; Condition (2) 0 min with 200 μL and 30 min with 400 μL; Condition (3) 0 min with 120 μL and 30 min 480 μL; Condition (4) 0 min with 120 μL and 24 h with 480 μL. The fluorescence properties of samples were tested under magnetic stirring within the experimental timescale of 60 min

Tuning the size of LSP *via* mechanical agitation. Upon dissolving SG7-LDH (4 mg) in 800 μL mixed solvent CH_3OH/TFA (1:3 v/v), the dynamic explorations of metastable LSP_{20} with ultrasound or without ultrasound were obtained by real-time CLSM ($\lambda_{\text{laser diode}}$ = 488 nm, λ_{PMT} = 500–680 nm) and time-dependent FL spectra. The effect of mechanical agitation on the size of LSP_{20} could be seen in SEM images by prolonging ultrasound time (50, 60 and 120 min).The FITS results were tested under magnetic stirring on F7000 (λ_{ex} = 450 nm, λ_{em} = 535 nm, V_{PMT} = 700 V). During ultrasound, the mixture of ice and water was used to keep a constant temperature at 0 ºC.

Preparation of SSP. SSP was prepared by mixing LSP_{seed} (2.5 mM) and $SG7_{agg}$ (2.5 mM) in equal volumes (1:1 v/v), followed with ultrasound for 1 h. Here, LSP_{seed} was fresh sonicated metastable LSPs. $SG7_{agg}$ was fresh stocking solution of SG7 in CH_3OH/TFA (1:3 v/v). The size of obtained SSP can be tuned by the concentration of LSP_{seed} or $SG7_{agg}$. SSPs (SSP_{20}, SSP_{50}, SSP_{100} and SSP_{3000}) were prepared by the same method mentioned above from fresh sonicated metastable LSP_{20}–LSP_{3000}. During ultrasound, the mixture of ice and water was used to keep a constant temperature at 0 ºC.

Regulation of the size of SSP_{20}. The concentration of LSP_{seed} (0.25, 2.5 and 5.0 mM) and $SG7_{agg}$ (1.0, 2.5 and 4.0 mM) or the volume ratio of LSP_{seed} and $SG7_{agg}$ (0.5:1, 1:1 and 2:1 v/v)

could be tuned to effectively regulate the size of SSP_{20}. Herein, LSP_{seed} (2.5 mM) was made from SG7-LDH (4 mg) in 800 μL CH_3OH/TFA (1:3 v/v) under ultrasound for 1 h, and LSP_{seed} with the other concentrations could be achieved by tuning the dosage of SG7-LDH. During ultrasound, the mixture of ice and water was used to keep a constant temperature at 0 ºC.

Living supramolecular polymerization of SSP_{20}. Fresh sonicated metastable LSP_{20} with different initial concentrations (0.25, 0.625, 1.25 and 2.50 mM) were respectively mixed with $SG7_{agg}$ (2.50 mM) to monitor the self-replication rates of LSP_{20} in seed-induced polymerization. In particular, real-time absorbance test of SSP_{20} herein was prepared without ultrasound during seed-induced polymerization because its slower kinetics benefits to achieve more details.

Living nature of seed-induced polymerization based on fresh metastable LSP_{20}. The product in Cycle 1 was prepared by mixing equal volumes of LSP_{seed} (2.5 mM) and $SG7_{agg}$ (2.5 mM) with ultrasound for 1 h. The products in Cycle 2 and Cycle 3 were made from the further addition of $SG7_{agg}$ (2.5 mM) into the products of last cycle (1:1 v/v) to repeat seed-induced supramolecular polymerization. All samples of SSP were freshly made for SEM. During ultrasound, the mixture of ice and water was used to keep a constant temperature at 0 ºC.

Chiral recognition of metastable LSM_{3000} to L- or D-Arg. The metastable LSM were firstly prepared by dissolving SG7-LDH (4 mg) into CH_3OH/TFA (5:3 v/v) (320 μL). The chiral recognition products were obtained by adding L- or D-Arg in TFA (100 mM, 680 μL) with ultrasound for different periods of time: 10 min for metastable LSM_{20}, 15 min for metastable LSM_{50}, 30 min for metastable LSM_{100} and 50 min for metastable LSM_{3000}. Until the end of ultrasound, polymer-L_{20} and polymer-D_{20} were generated almost at the same time, so did polymer-L_{50} and polymer-D_{50}. Both of polymer-L_{100} and polymer-L_{3000} took precedence over

generating their polymer-D. The fluorescent difference of chiral recognition products for LSM_{3000} to L- or D-Arg could be seen in CLSM images ($\lambda_{laser\ diode}$ = 405 nm and λ_{PMT} = 420–580 nm for blue-light metastable LSM, $\lambda_{laser\ diode}$ = 488 nm and λ_{PMT} = 500–680 nm for yellow-light polymer). FL spectra were used to precisely test the existence of normally elongated LSP (yellow light at 530–545 nm), metastable LSM (blue light at ~480 nm) and polymer-L/D (525–530 nm). During ultrasound, the mixture of ice and water was used to keep a constant temperature at 0 ºC.

Universality experiments. All monomers solutions of 12.5 mM at pH = 8.00 were prepared, including BSA, 3-ABSA, 2,5-DABSA, CR and DHNS, respectively. These molecules were intercalated into LDHs respectively under the same conditions as SG7-LDH by adding 0.313 g of Mg_3Al-Cl-LDH into MSS solutions. The products were washed with deionized water and CH_3OH respectively for three times and re-dispersed in CH_3OH (20.0 g L^{-1}) for further use. Corresponding metastable LSP products or LSP_{seed} were prepared by dissolving as-prepared intercalated LDH (4 mg) in CH_3OH/TFA (1:3 v/v) (800 μL) with ultrasound for 1 h. During ultrasound, the mixture of ice and water was used to keep a constant temperature at 0 ºC.

The products in Cycle 1 from 3-ABSA were prepared by mixing equal volumes of LSP_{seed} (2.5 mM) and 3-ABSA solution in CH_3OH/TFA (1:3 v/v) (2.5 mM) with ultrasound for 1 h. The products in Cycle 2 are made from further addition of 3-ABSA solution (2.5 mM) into the products in Cycle 1 (1:1 v/v) to repeat seed-induced supramolecular polymerization. The products in Cycle 1 and 2 from 2,5-DABSA were prepared by the same method.

Sample preparation. In order to avoid the severe aggregation during preparing the samples for SEM imaging, the PSS (poly(sodium-p-styrenesulfonate)) modified silicon substrate was used. Once the modified substrate is immersed in the fresh LSM solution, the sufficient $-SO_3^-$ of PSS

will form $-SO_3H$, which is beneficial to form H-bond between PSS and LSM. Moreover, the immersing time of substrate should be as short as possible to avoid too many LSM dip-coated on the modified substrate.

Model construction. In order to better understand the mechanism of living assembly for supermolecular polymers, density functional theory (DFT) calculations were carried out. The SG7 was taken to investigate as a model molecule. First of all, a model representing SG7 monomer was built by dispersing eight SG7 molecules in solvent ([SG7] = 2.5 mM in CH_3OH/TFA (5:3 v/v).

After that, a series of models were constructed, representing SG7 intercalated MgAl-layered double hydroxide (SG7-LDH), SG7 LSM with different orientation (SG7-θ, θ ranged from 0º to 90º), SG7 LSM binding SG7 molecule with θ = 35º, SG7 LSM binding SG7 molecule with θ = 9º, SG7 LSM binding D-Arg (LSM+D), and SG7 LSM binding L-Arg (LSM+L).

The model of Mg_3Al-SG7-LDH was built with the space group of $p\bar{3}m1$, indicating that the lattice parameters were $\alpha = \beta = 90º$, $\gamma = 120º$. The other three lattice parameters were referred to the experimental X-ray diffraction measurement, *i.e.*, $a = b = 3.0$ Å, $c = 9.7$ Å. The supercell of SG7-LDH was 16 × 4 × 1 in the *a*-, *b*-, and *c*- directions. The molar ratio of Mg to Al was 3 to 1, in accordance with the experiment. Four negative tetravalent SG7 anions were put in the MgAl-LDH interlayer gallery to balance the positive charge of LDH matrix. Therefore, the chemical formula of model MgAl-SG7-LDH was $Mg_{48}Al_{16}(OH)_{128}(C_{16}H_6O_{10}S_3)_4$. The model of MgAl-BSA-LDH, MgAl-ABSA-LDH, MgAl-DABSA-LDH, MgAl-DHNS-LDH, and MgAl-CR-LDH were built in the similar way with that of MgAl-SG7-LDH. The chemical formula of MgAl-BSA-LDH, MgAl-ABSA-LDH, MgAl-DABSA-LDH, MgAl-DHNS-LDH, and MgAl-CR-LDH was $Mg_{48}Al_{16}(OH)_{128}(C_6H_5SO_3)_{16}$, $Mg_{48}Al_{16}(OH)_{128}(C_6H_6NSO_3)_{16}$,

$Mg_{48}Al_{16}(OH)_{128}(C_6H_7N_2SO_3)_{16}$, $Mg_{48}Al_{16}(OH)_{128}(C_{10}H_5SO_5)_5Cl$, and $Mg_{48}Al_{16}(OH)_{128}(C_{32}H_{22}N_6S_2O_6)_2(NO_3)_{12}$, respectively.

A series of SG7 LSM models with different orientation were constructed. The orientation angle θ, was defined as the inclined angle between the principal axis and the elongation axis of SG7 LSM, as displayed in Supplementary Figure 11. The orientation angle θ ranged from 0° to 90° with the step of 1°. For the computational cost-effectiveness, each LSM contained 8 SG7 molecules. Furthermore, the effect of the number of SG7 molecules is investigated by building LSM containing 4 SG7 molecules.

The models of SG7 LSM+SG7 monomer (θ = 35°), SG7 LSM+SG7 monomer (θ = 9°), LSM+D and LSM+L were constructed by combining SG7 LSM with another SG7 molecule with θ = 35°, or θ = 9°, and combining SG7 LSM with D-Arg, or L-Arg, respectively.

Computational methods. All the calculations in this work were performed with the DMol^3 code in Accelrys materials studio version 5.5 software packages (Accelrys Software Inc., San Diego, CA).[1,2] The calculations were carried out in the DFT framework with the generalized gradient approximation (GGA) Perdew-Burke-Ernzerhof (PBE) as the exchange-correlation functional.[3] The DFT dispersion correction was dealt with the Tkatchenko-Scheffler method to describe the noncovalent forces.[4] The basis set was set as the double-numerical plus polarization (DNP) for expanding the Kohn-Sham orbitals and improving the accuracy of calculations. In the process of geometry optimization, three convergence criteria were used as follows: (1) energy tolerance of 2.0×10^{-5} hartree, (2) force tolerance of 1.0×10^{-3} hartree/Å, (3) displacement tolerance of 5.0×10^{-3} Å.

The binding energy (E_B) between SG7 LSM and another SG7 molecule was calculated with

Equation (1):

$$E_{\mathrm{B}} = E_{\mathrm{SG7\ LSM\ +\ SG7\ molecule\ complex}} - E_{\mathrm{SG7\ LSM}} - E_{\mathrm{SG7\ molecule}} \qquad (1)$$

where $E_{\mathrm{SG7\ LSM\ +\ SG7\ molecule\ complex}}$, $E_{\mathrm{SG7\ LSM}}$, and $E_{\mathrm{SG7\ molecule}}$ were the energies of corresponding subscripts. The binding energy between SG7 LSM and Arg was calculated in the similar way.

In order to uncover the orientation of simple-structured molecules in MgAl-LDH interlayer, *ab initio* molecular dynamics (AIMD) simulations were performed on MgAl-SG7-LDH, MgAl-BSA-LDH, MgAl-ABSA-LDH, MgAl-DABSA-LDH, MgAl-DHNS-LDH, and MgAl-CR-LDH. The AIMD simulations were performed in isothermal-isobaric (*NPT*) ensemble, with the temperature of 298.15 K and the pressure of 0.1 MPa. The temperature and pressure controls were accomplished using the Andersen method[5] and the Berendsen method[6], respectively. A total simulation time of 100 ps was performed for each model with a time step of 1 fs.

2. Supplementary Figures

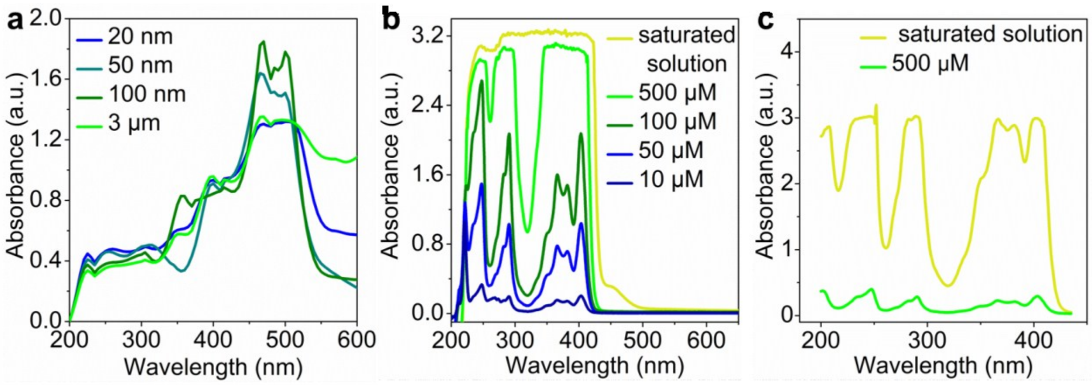

Supplementary Figure 1. UV-vis spectra of **(a)** SG7-LDH aqueous dispersion (20.0 g L^{-1}) with different sizes (optical path = 0.2 mm), **(b)** SG7 solution in CH_3OH with a set of concentrations (optical path = 10 mm) and **(c)** SG7 solution in CH_3OH with different concentrations (optical path = 0.2 mm).

It is worth noting that instrument range of U-3900H is −5.5~5.5 Abs. However, the absorbances of samples are too high to be tested in the common quartz cuvette (optical path = 10 mm), such as SG7-LDH and SG7 solution with high concentrations in our work. Therefore, the special quartz cuvette (optical path = 0.2 mm) is chosen for the precise test of these samples to avoid the overrange absorbance.

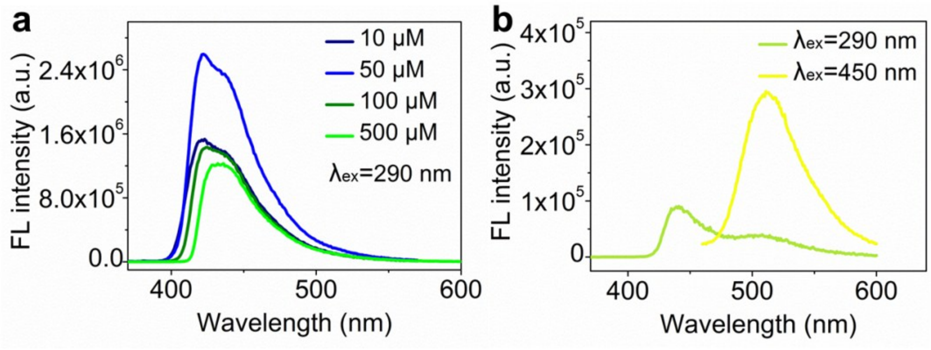

Supplementary Figure 2. FL emission spectra of SG7 solution in CH_3OH: **(a)** with a set of concentrations and **(b)** saturated solution.

SG7 is a kind of typical optical molecule with quite big difference in fluorescence properties between monomer and excimer, providing the prerequisite for naked eye visualization under UV lamp, which can be confirmed by above experimental data. In FL spectra, SG7 monomer dissolved in CH_3OH has one main peak at λ = 420~435 nm (Supplementary Fig. 2). When the concentration of SG7 in CH_3OH approaches saturation, the distance between molecules is shorter than 10 Å, the emission of excimer state will appear at a red-shifted wavelength (λ_{em} = 511 nm) than that of monomer.[5] As shown in UV and FL spectra, SG7 solution showed red shift with the increase of concentration, attributing to its increased J-aggregate structure and decreased molecular distance (Supplementary Fig. 1−2). The above phenomena provide the prerequisite for naked eye visualization of assembly events.

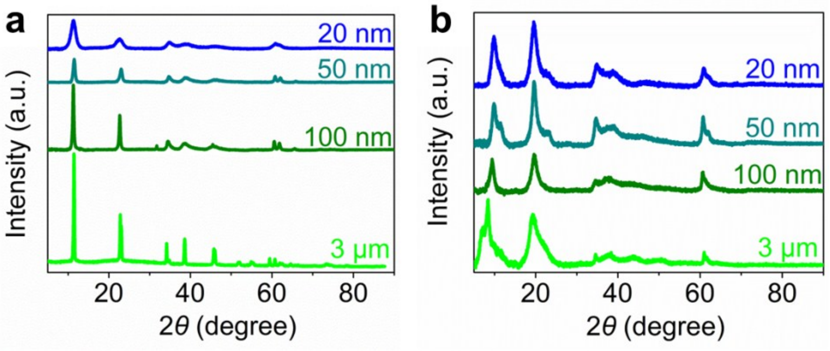

Supplementary Figure 3. XRD patterns of **(a)** pure LDH precursor powder and **(b)** SG7-LDH with different sizes.

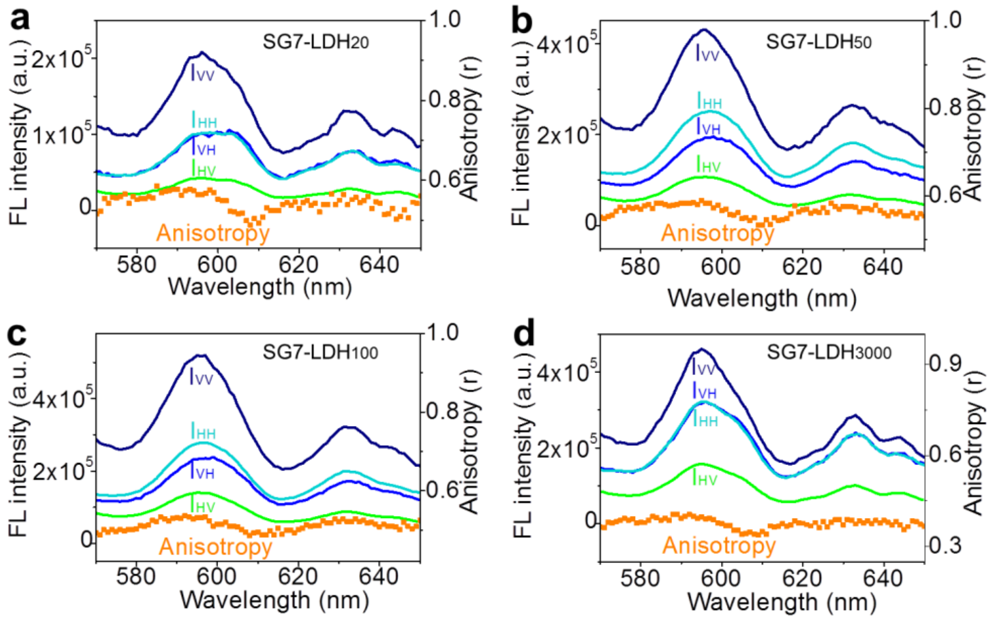

Supplementary Figure 4. Polarized FL profiles and anisotropic value (*r*) for **(a)** SG7-LDH$_{20}$ (r = 0.565), **(b)** SG7-LDH$_{50}$ (r = 0.589), **(c)** SG7-LDH$_{100}$ (r = 0.531) and **(d)** SG7-LDH$_{3000}$ (r = 0.398) in solid state on the quartz plate, respectively.

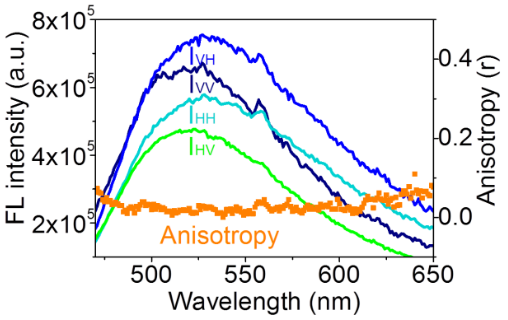

Supplementary Figure 5. Polarized FL profiles and anisotropic value (*r*) for untreated SG7 powder (*r* = 0.0201).

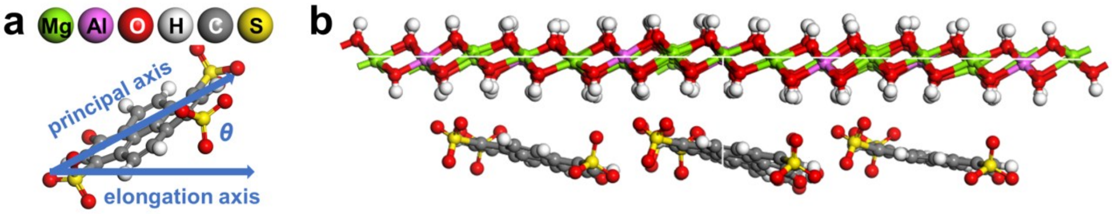

Supplementary Figure 6. (a) Schematic illustration for the definition of orientation angle, θ. **(b)** The snapshot of SG7-LDH after AIMD simulations of 100 ps.

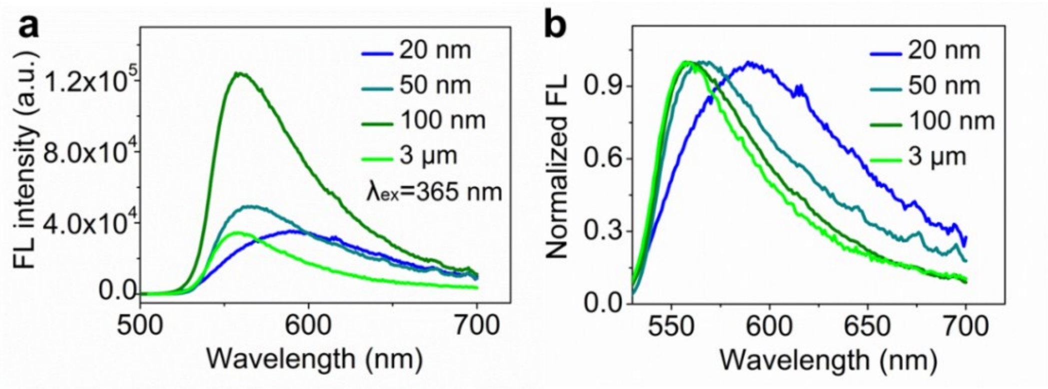

Supplementary Figure 7. (a) FL emission spectra and **(b)** corresponding normalized FL spectra of SG7-LDH with different sizes in CH_3OH.

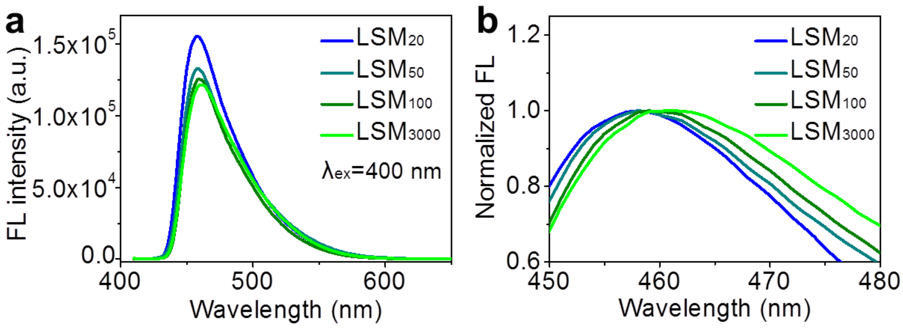

Supplementary Figure 8. (a) FL emission spectra and **(b)** corresponding normalized FL spectra of metastable LSM_{20}–LSM_{3000} after removal of LDH layers in CH_3OH/TFA (5:3 v/v).

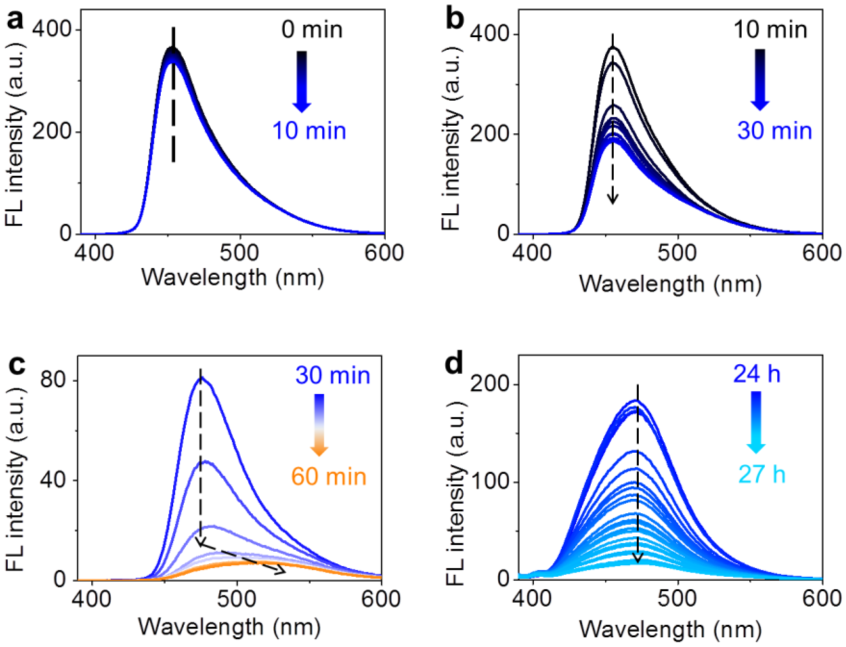

Supplementary Figure 9. FL spectra of metastable LSM_{20} in CH_3OH/TFA (5:3 v/v) within **(a)** 0−10 min and **(b)** 10−30 min. FL spectra of the transformation of metastable LSM_{20} in CH_3OH/TFA (1:3 v/v), after keeping metastable LSM_{20} in CH_3OH/TFA (5:3 v/v) for **(c)** 30 min and **(d)** 24 h.

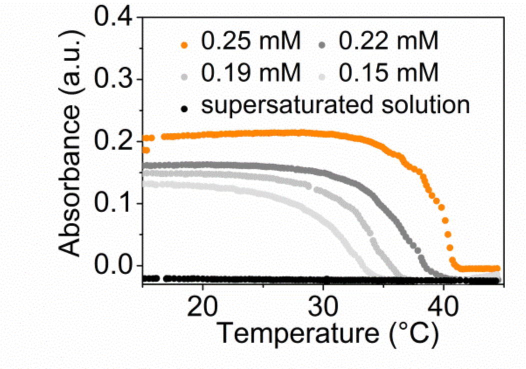

Supplementary Figure 10. Temperature-dependent absorption of metastable LSP with a set of low concentration and supersaturated SG7 solution in CH_3OH/TFA (1:3 v/v) (5 mM) at 475 nm in UV-vis spectra.

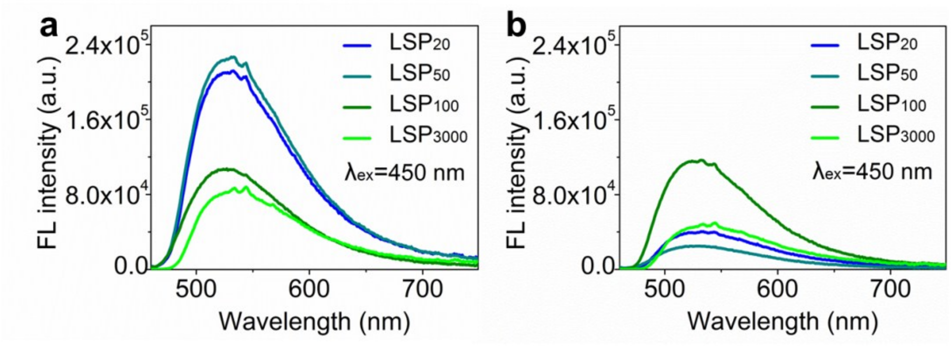

Supplementary Figure 11. FL spectra of LSP_{20}–LSP_{3000}: **(a)** freshly sonicated metastable LSP and **(b)** elongated LSP (12 h) after ultrasound.

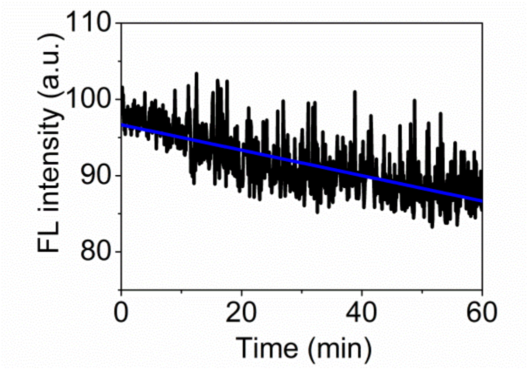

Supplementary Figure 12. FITS of freshly sonicated LSP_{20} under magnetic stirring at 535 nm.

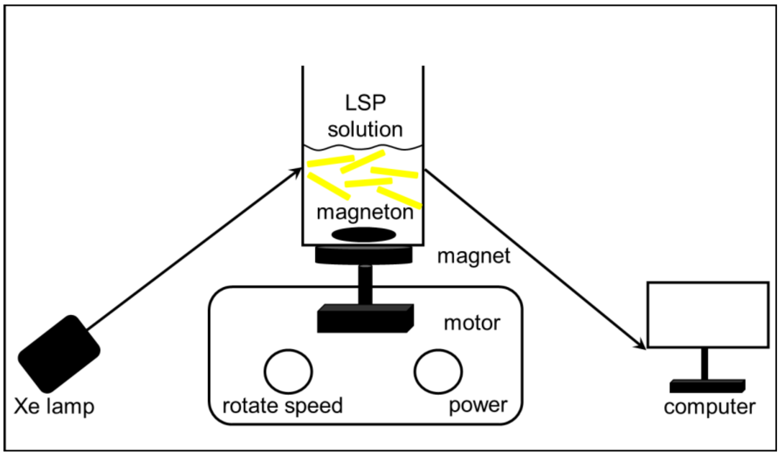

Supplementary Figure 13. Schematic presentation of experimental set-up of FITS under magnetic stirring.

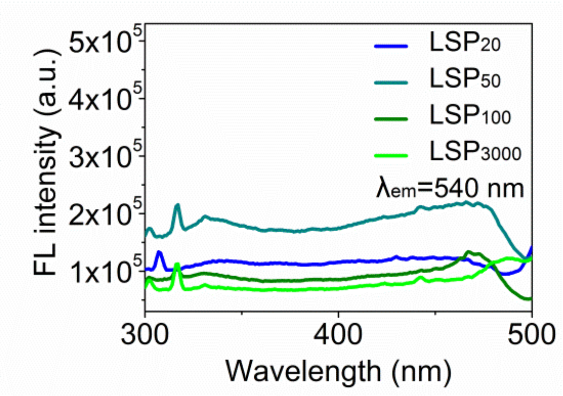

Supplementary Figure 14. FL excitation spectra of LSP_{20}–LSP_{3000}.

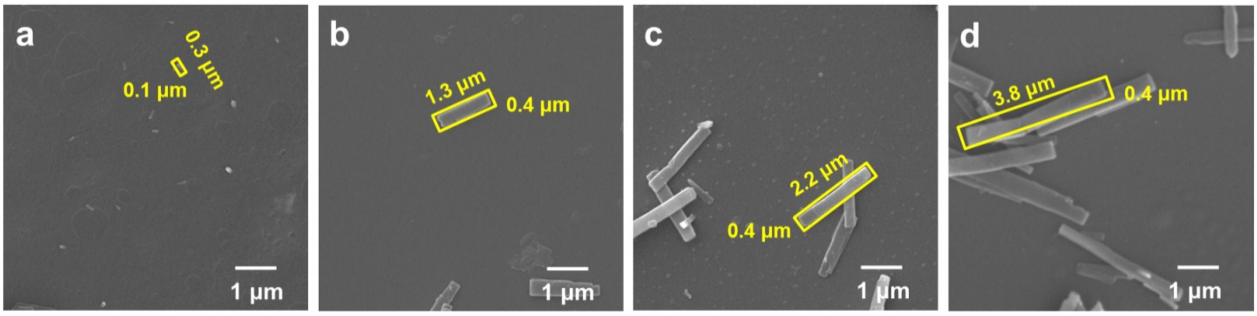

Supplementary Figure 15. SEM images of LSP_{20} with ultrasound elongated for different time: **(a)** 0 h, **(b)** 1 h, **(c)** 3 h and **(d)** 12 h.

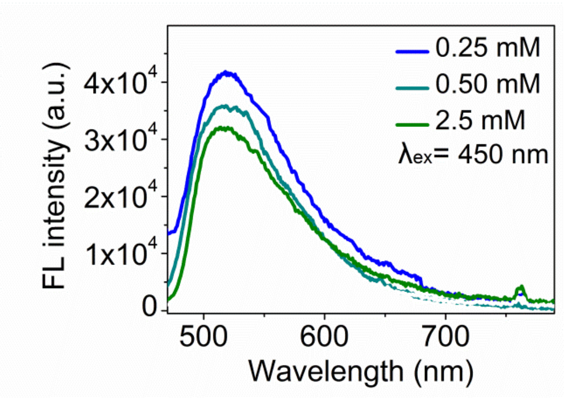

Supplementary Figure 16. FL spectra of fresh SSP_{20} made from LSP_{seed} with different concentrations.

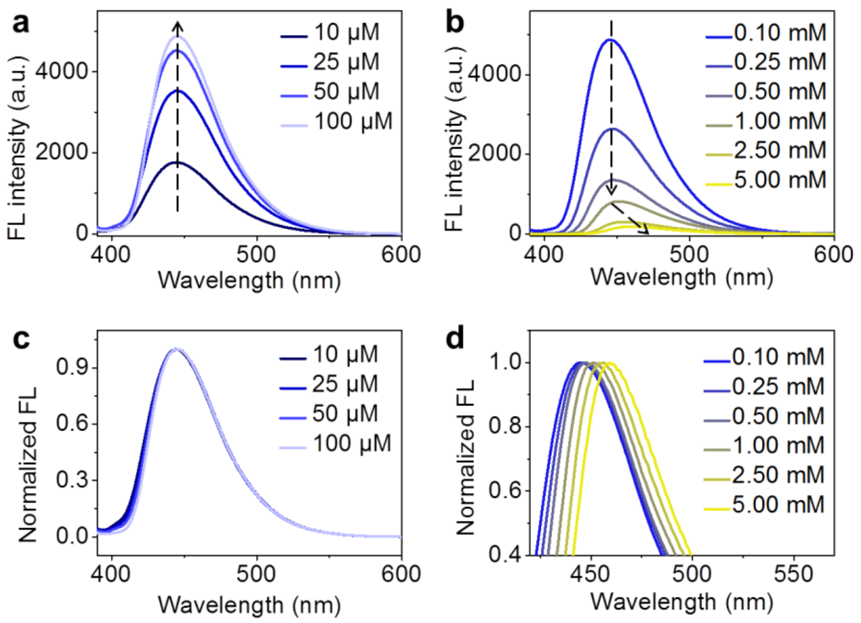

Supplementary Figure 17. FL spectra SG7 powder dispersed in CH_3OH/TFA (1:3 v/v). **(a)** 10−100 μM SG7 with increased intensity. **(b)** 0.1−5 mM SG7 with decreased intensity and red-shifted wavelength. **(c−d)** Normalized FL spectra of a−b, respectively.

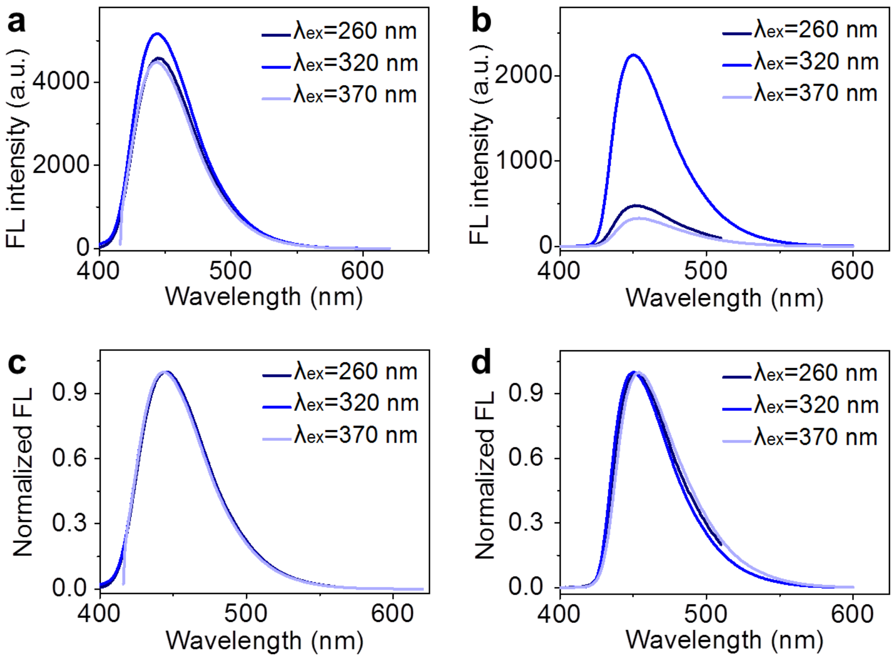

Supplementary Figure 18. FL spectra of control sample: SG7 powder dispersed in CH_3OH/TFA (1:3 v/v). **(a)** 0.0625 mM SG7 (named as SG7 monomer, λ_{em}=445 nm). **(b)** 2.5 mM SG7 (named as $SG7_{agg}$, λ_{em}=450 nm). **(c−d)** Normalized FL spectra of a and b, respectively.

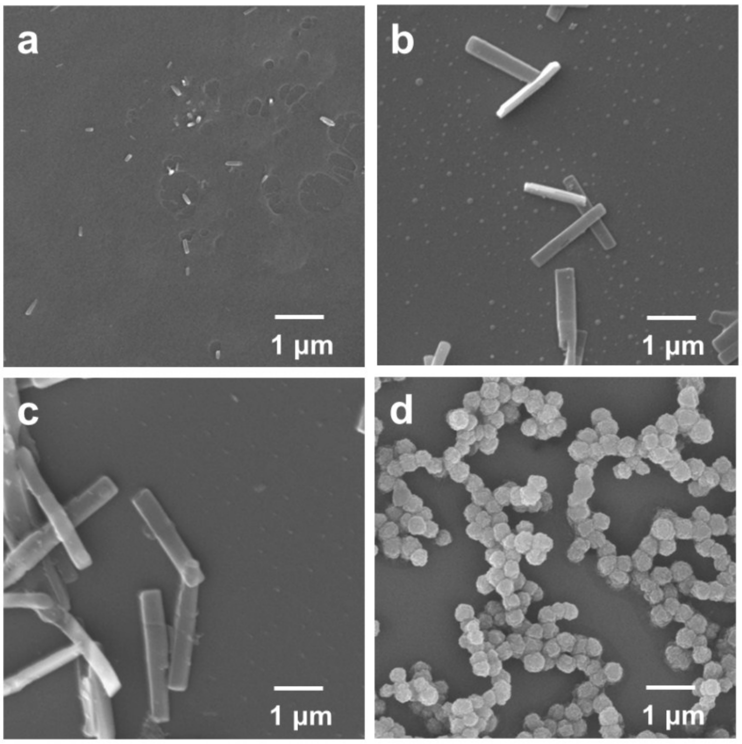

Supplementary Figure 19. SEM images of **(a)** metastable LSP made of SG7, corresponding fresh SSPs in **(b)** Cycle 1 and **(c)** Cycle 2, and **(d)** contrast sample: physically mixing pure SG7 and Cl-LDH in CH_3OH/TFA (1:3 v/v) with the same dosage as LSP.

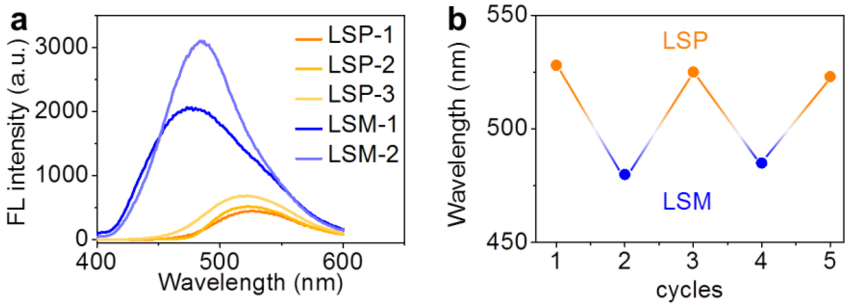

Supplementary Figure 20. FL spectra of the reversible disassembly (λ_{em} = 480−490 nm) and re-assembly (λ_{em} = 525 nm) of LSP_{20}.

Compared with crystals, the significant feature of supramolecular polymer is reversibility. For our system, the reversibility can be proved by disassembly (in CH_3OH/TFA (1:2 v/v)) and re-assembly (in CH_3OH/TFA (1:3 v/v)) of LSP (Supplementary Fig. 20). FL spectra are used to monitor this process. By increasing the ratio of CH_3OH to CH_3OH/TFA (1:2 v/v), LSP (λ_{em} = 525 nm) disassembled to LSM (λ_{em} = 490 nm), as confirmed by FL wavelength shift from 525 to 490 nm; by decreasing the ratio of CH_3OH to CH_3OH/TFA (1:3 v/v), LSM could quickly re-assemble to LSP, as confirmed by FL wavelength shift from 490 to 525 nm (λ_{em} = 370 nm, Supplementary Fig. 20).

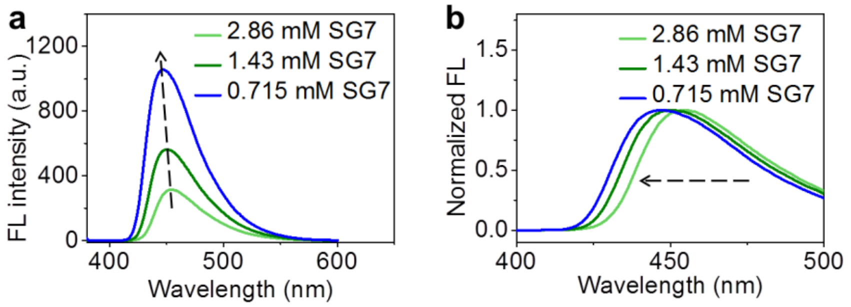

Supplementary Figure 21. (a) FL spectra and **(b)** normalized FL of SG7 solution (0.715 mM SG7 powder dispersed in CH_3OH/TFA (1:2.5 v/v)).

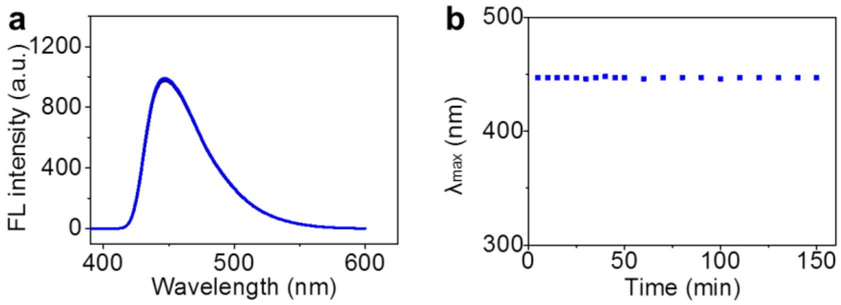

Supplementary Figure 22. (a) FL spectra and **(b)** time course of maximum emission wavelength of SG7 solution (0.715 mM SG7 powder dispersed in CH_3OH/TFA (1:2.5 v/v)).

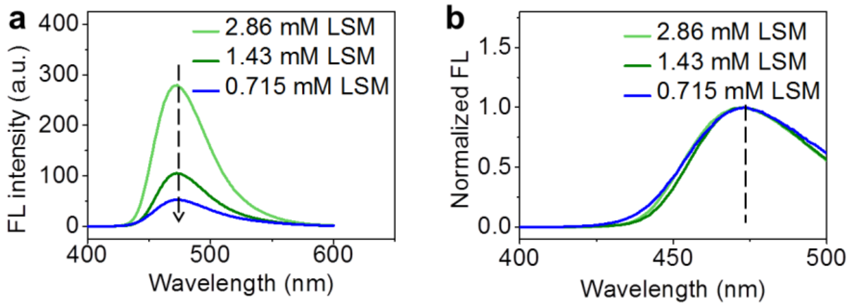

Supplementary Figure 23. (a) FL spectra and **(b)** normalized FL of diluted LSM in CH_3OH/TFA (1:2.5 v/v).

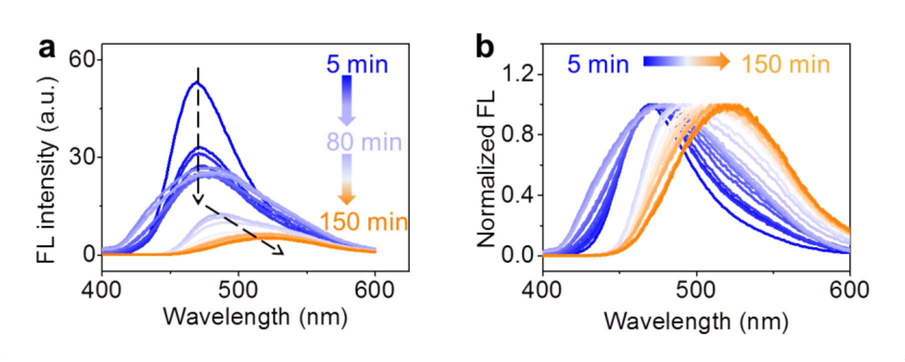

Supplementary Figure 24. **(a)** FL spectra and **(b)** normalized FL of the transformation from metastable LSM_{20} to metastable LSP_{20} in CH_3OH/TFA (1:2.5 v/v) ([SG7] = 0.715 mM).

In detail, the dilution to LSM had no effect on the transformation from metastable LSM to metastable LSP, which can be confirmed by FL spectra. During dilution, the constant wavelength indicated no disassembly. It should be noted that the decreased FL intensity resulted from dilution (Supplementary Fig. 23). For diluted LSM, it can finally transform to LSP, confirmed by red shift to 525 nm (Supplementary Fig. 24). In contrast, the $SG7_{agg}$ disassembled to SG7 monomer after dilution and no LSP was formed, confirmed by blue shift of $SG7_{agg}$ and absence the emission at 525 nm (Supplementary Fig. 21−22).

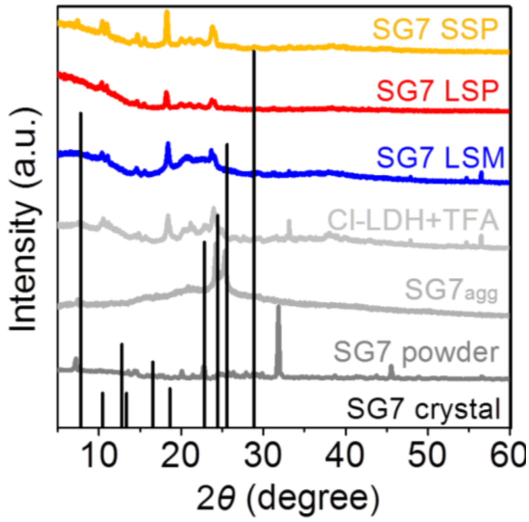

Supplementary Figure 25. XRD patterns of SG7 SSP (orange), SG7 LSP (red), SG7 LSM (blue), destroyed Cl-LDH in TFA (light gray), $SG7_{agg}$ (gray), SG7 powder (dark gray) and SG7 crystal (black).

None of products in our system is crystal. This can be confirmed by comparing the XRD results (Supplementary Fig. 25), including metastable LSM, LSP, SSP, untreated SG7 powder, treated SG7 powder in CH_3OH/TFA (1:3 v/v) ($SG7_{agg}$), Cl-LDH dissolved in TFA and SG7 crystal (JCPDS data base card No: 42-1955). Among all samples, only the XRD pattern of $SG7_{agg}$ shows two peaks in line with SG7 crystal. The XRD pattern of LSM shows several weak peaks, which are attributed to the metal salts of the LDH dissolved in TFA. Thus, the SG7 crystal did not appear.

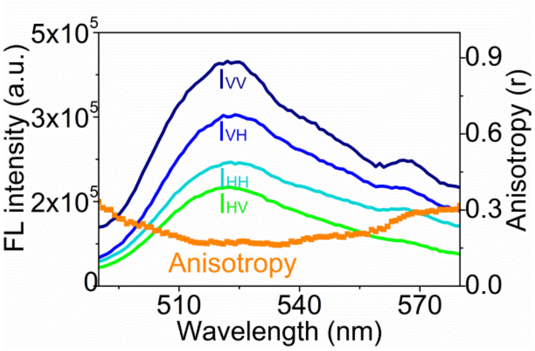

Supplementary Figure 26. Polarized FL profiles and anisotropic value (*r*) for SG7-LDH_{20}-surface ($r = 0.171$) in solid state on the quartz plate.

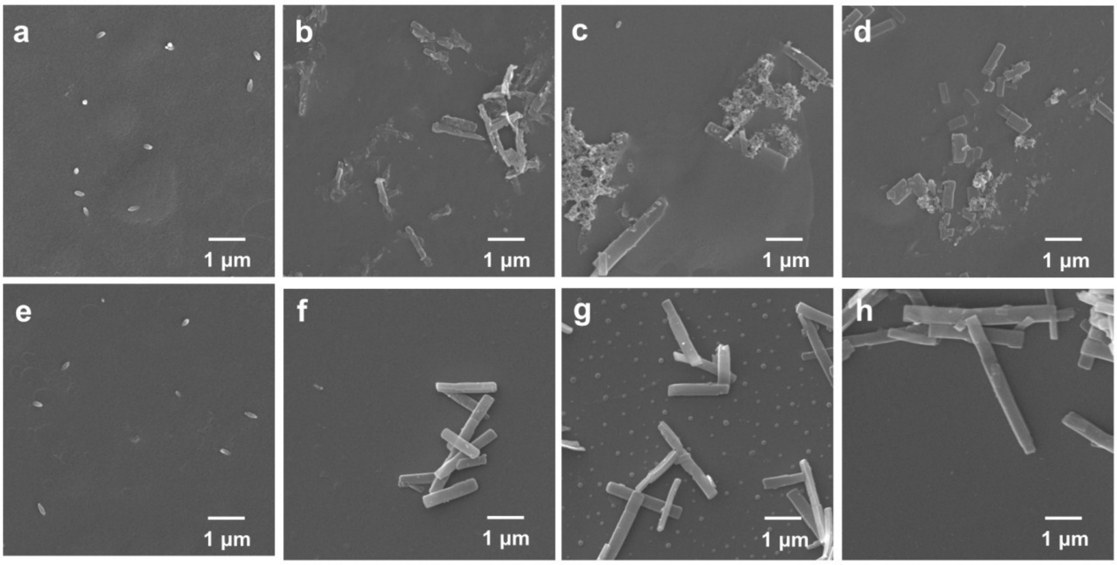

Supplementary Figure 27. SEM images of **(a−d)** SP_{20} made from SG7-LDH_{20}-surface and corresponding SSP in Cycle 1−3, **(e−h)** mixed SP_{20} made from physically mixed SG7-LDH_{20}-surface and SG7-LDH_{20} in equal volumes and corresponding SSP in Cycle 1−3.

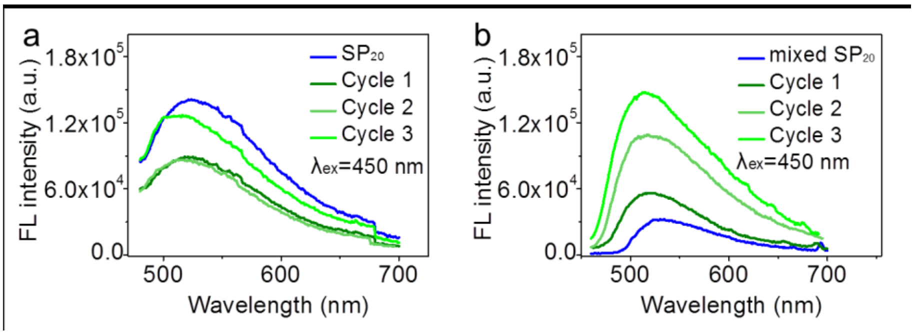

Supplementary Figure 28. FL spectra of **(a)** SP_{20} made from SG7-LDH-surface and corresponding SSP in Cycle 1−3 **(b)** mixed SP_{20} made from physically mixed SG7-LDH_{20}-surface and SG7-LDH_{20} in equal volumes and corresponding SSP in Cycle 1−3.

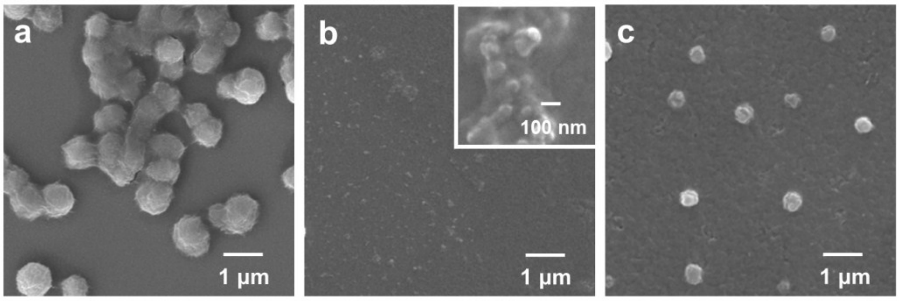

Supplementary Figure 29. SEM images of **(a)** pure SG7 **(b)** pure Cl-LDH and **(c)** SG7+LDH dissolved in CH_3OH/TFA (1:3 v/v), all of them with the same dosage as SG7 LSP.

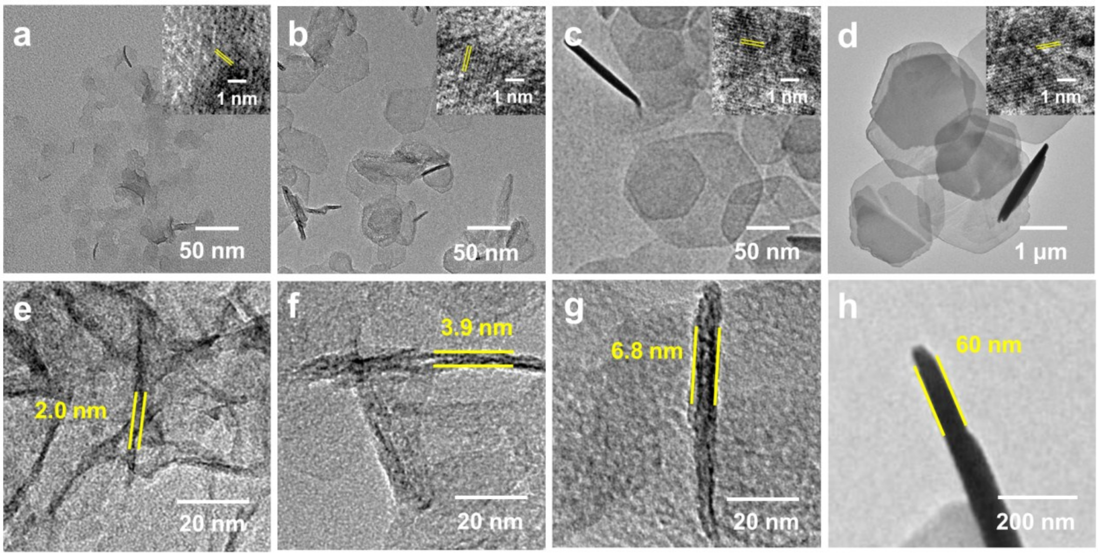

Supplementary Figure 30. TEM images of pure LDH precursors with different sizes: **(a)** 20 nm, **(b)** 50 nm, **(c)** 100 nm and **(d)** 3 μm (insets: corresponding lattice fringes). **(e−h)** HRTEM images for the thickness of LDH in a−d, respectively.

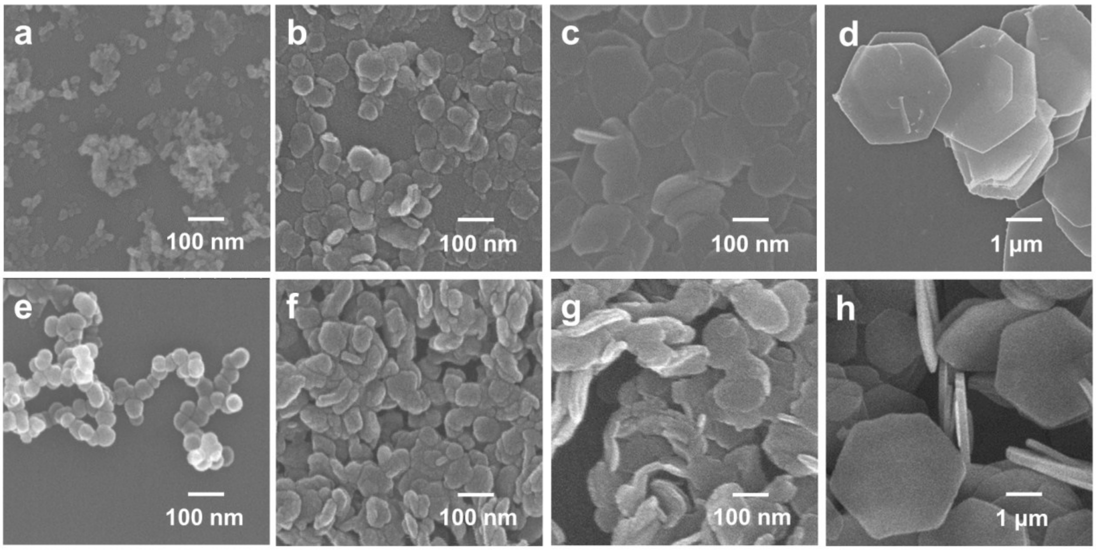

Supplementary Figure 31. SEM images of pure LDH precursors with different sizes: **(a)** 20 nm, **(b)** 50 nm, **(c)** 100 nm and **(d)** 3 μm. **(e−h)** SEM images of SG7-LDH by ion-exchange of LDHs in a−d, respectively.

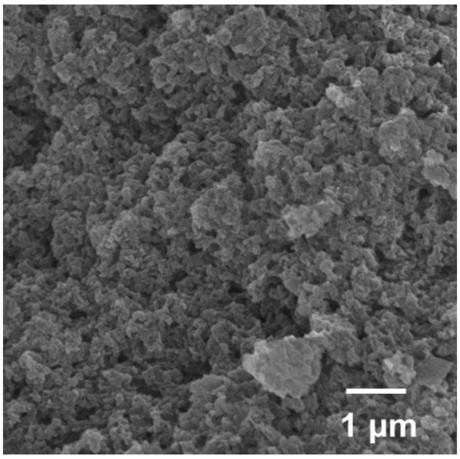

Supplementary Figure 32. SEM images of SG7 LSM_{20} access to corresponding equilibrium state in CH_3OH/TFA (5:3 v/v).

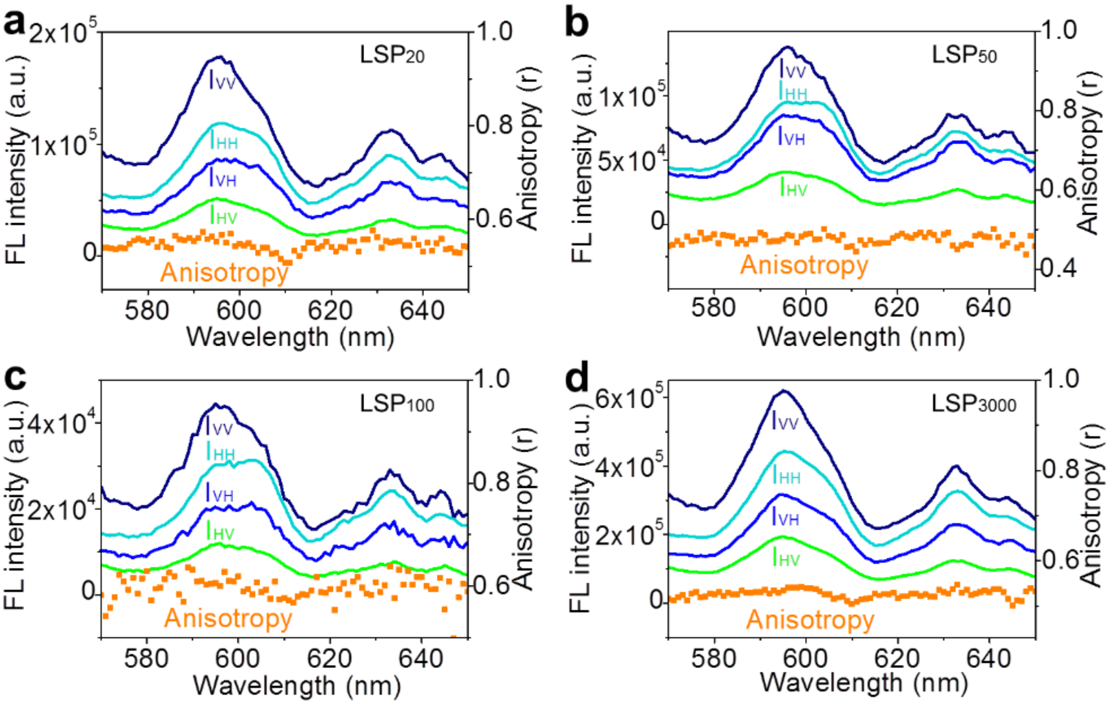

Supplementary Figure 33. Polarized FL profiles and anisotropic value (r) for **(a)** LSP_{20} (r = 0.552), **(b)** LSP_{50} (r = 0.476), **(c)** LSP_{100} (r = 0.606) and **(d)** LSP_{3000} (r = 0.535) in solid state on the quartz plate, respectively.

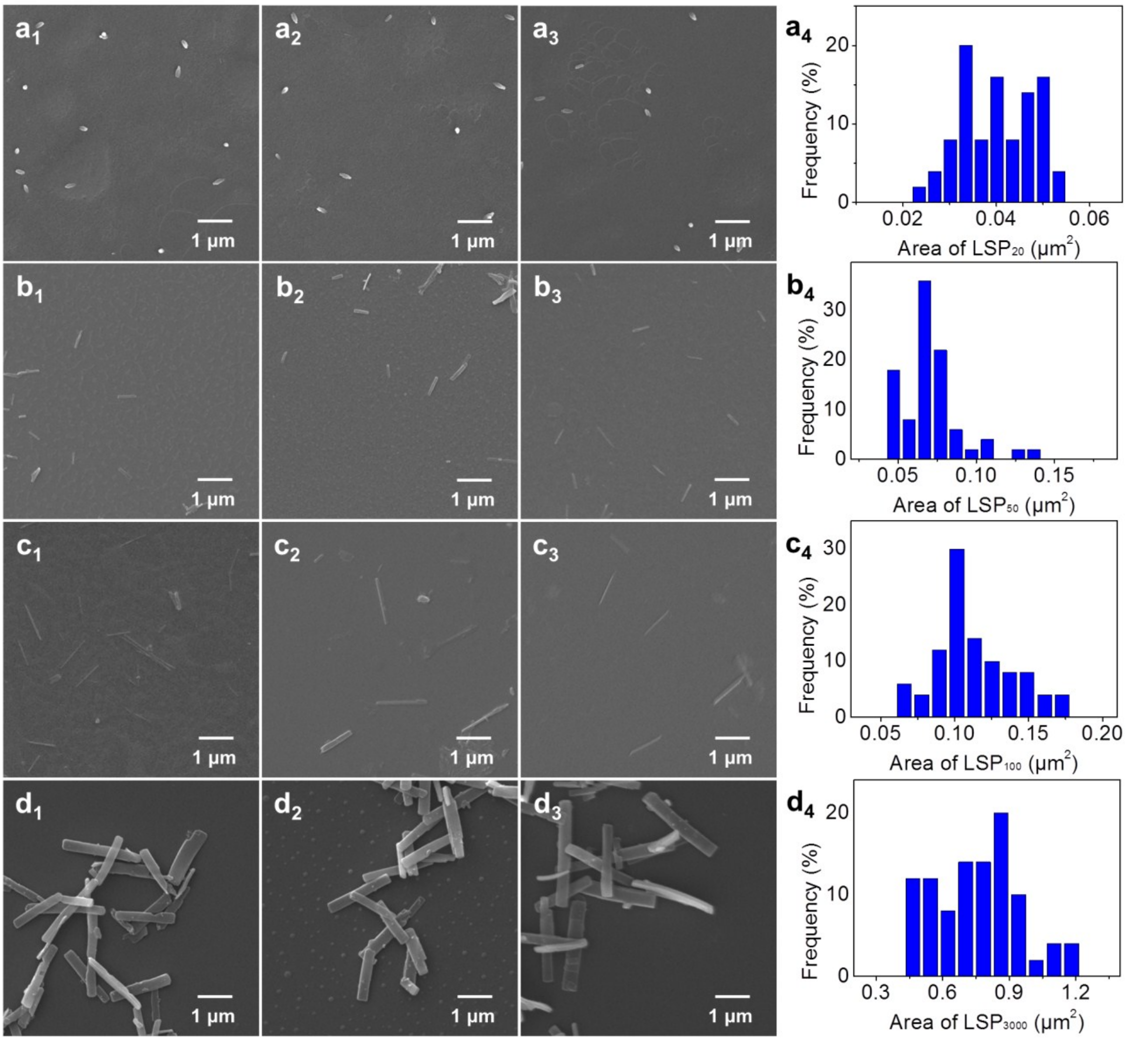

Supplementary Figure 34. SEM images of **(a_1–a_3)** metastable LSP_{20}, **(b_1–b_3)** metastable LSP_{50}, **(c_1–c_3)** metastable LSP_{100} and **(d_1–d_3)** metastable LSP_{3000} in batch 1-3, respectively. **(a_4–d_4)** Area distribution of LSP_{20}–LSP_{3000}, respectively, which was obtained by evaluating over 50 objects in corresponding SEM images.

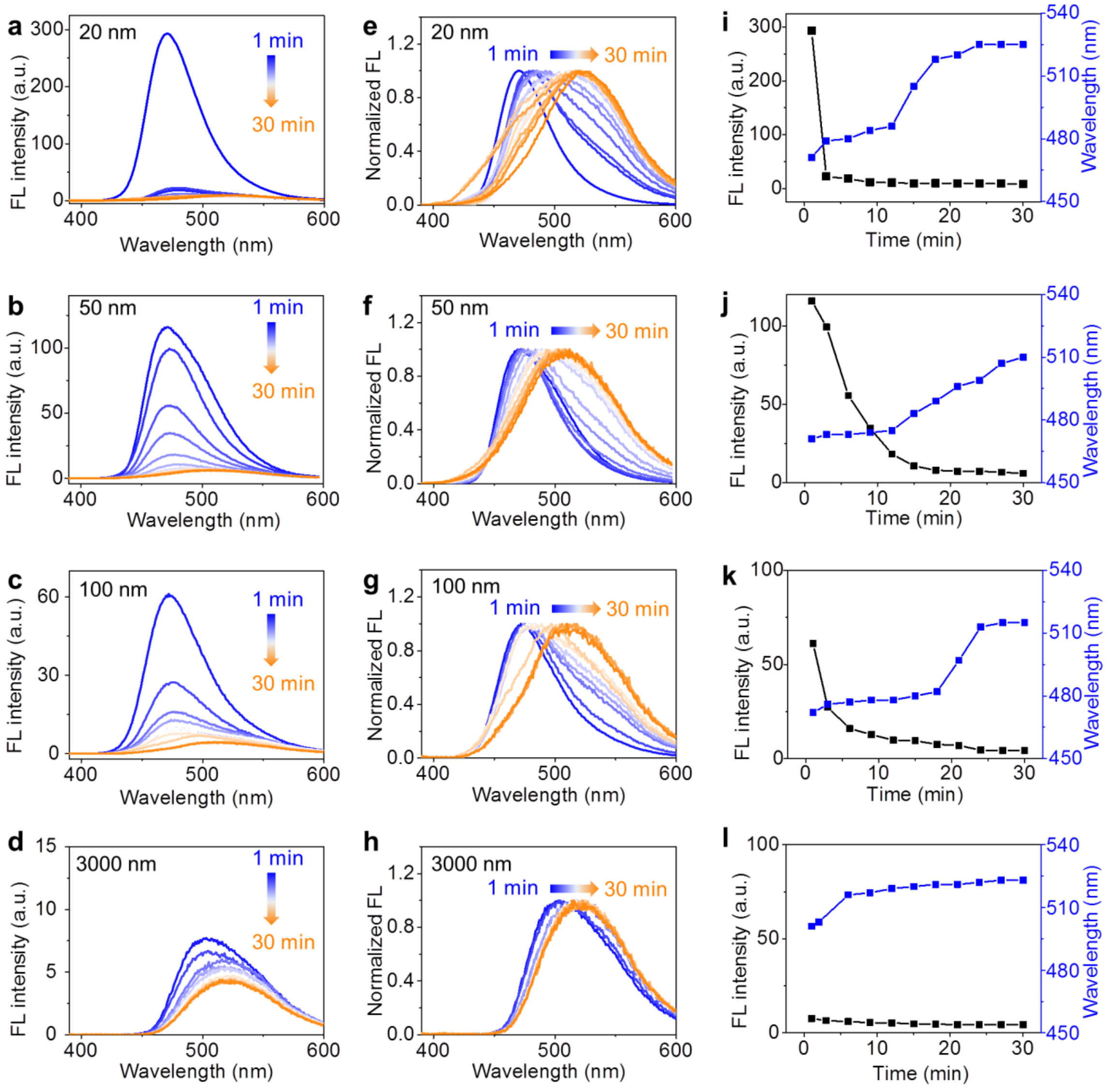

Supplementary Figure 35. FL spectra of transformation from metastable **(a)** LSM_{20}; **(b)** LSM_{50}; **(c)** LSM_{100} and **(d)** LSM_{3000} to corresponding LSP in CH_3OH/TFA (1:3 v/v). **(e−h)** Normalized FL spectra in a−d, respectively. **(i−l)** Time-dependent FL intensity and wavelength in a−d, respectively.

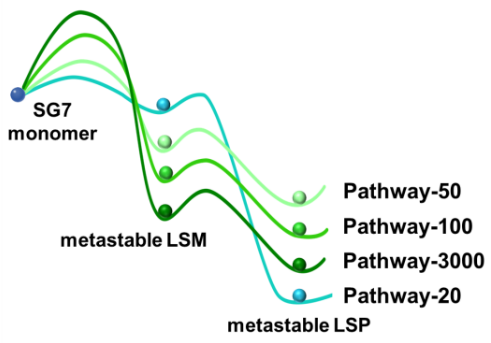

Supplementary Figure 36. Schematic illustration of pathway for the formation of metastable LSP_{20}–LSP_{3000}.

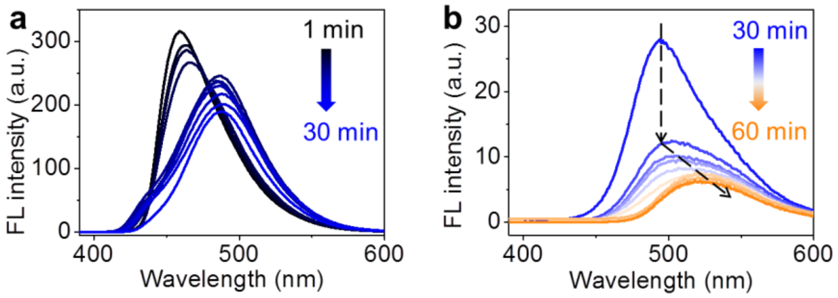

Supplementary Figure 37. (a) FL spectra of metastable LSM_{20} in CH_3OH/TFA (1:1 v/v). **(b)** FL spectra of metastable LSM_{20} from (a), transforming to metastable LSP_{20} in CH_3OH/TFA (1:3 v/v).

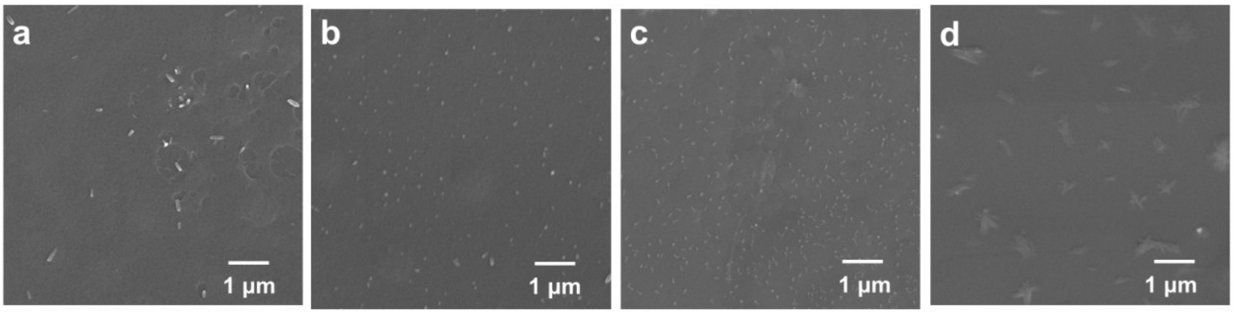

Supplementary Figure 38. SEM images of **(a−c)** metastable LSP_{20} prepared from Condition (1)−(3), respectively and **(d)** failed metastable LSP_{20} of Condition (4).

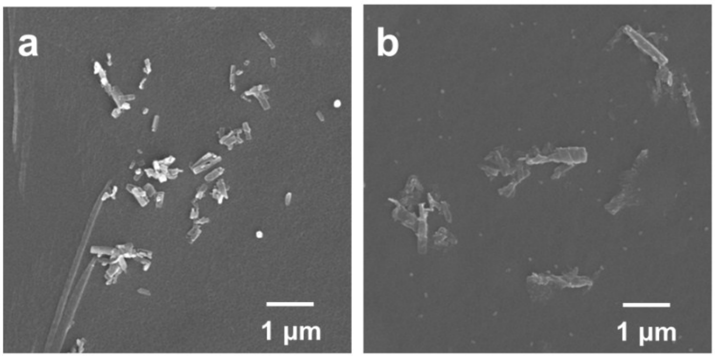

Supplementary Figure 39. SEM image of LSP_{20} without ultrasound kept for **(a)** 1 h and **(b)** 12 h.

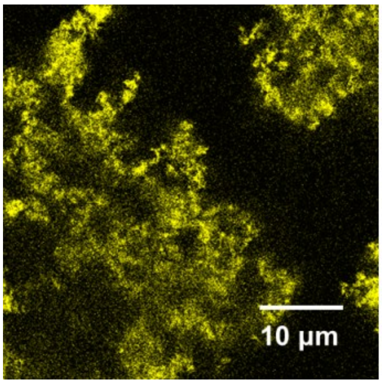

Supplementary Figure 40. CLSM images of metastable LSP_{20} without ultrasound.

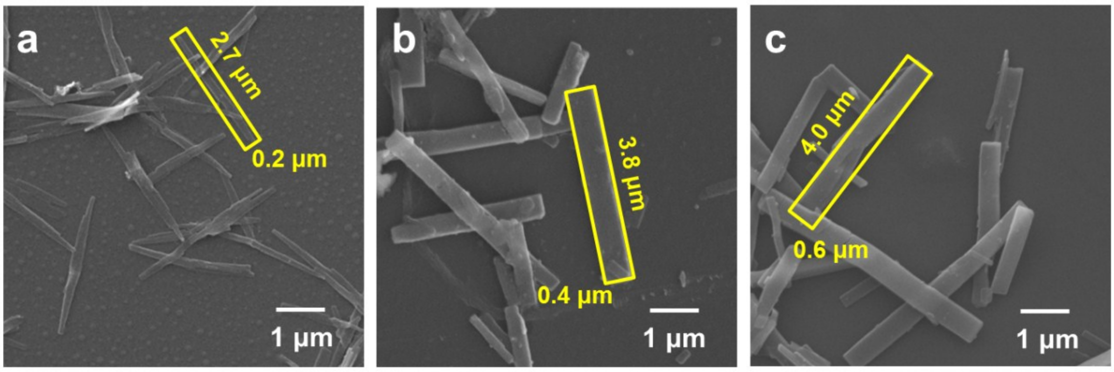

Supplementary Figure 41. SEM images of LSP_{20} elongated for 12 h with different ultrasound time: **(a)** 50 min, **(b)** 60 min and **(c)** 120 min.

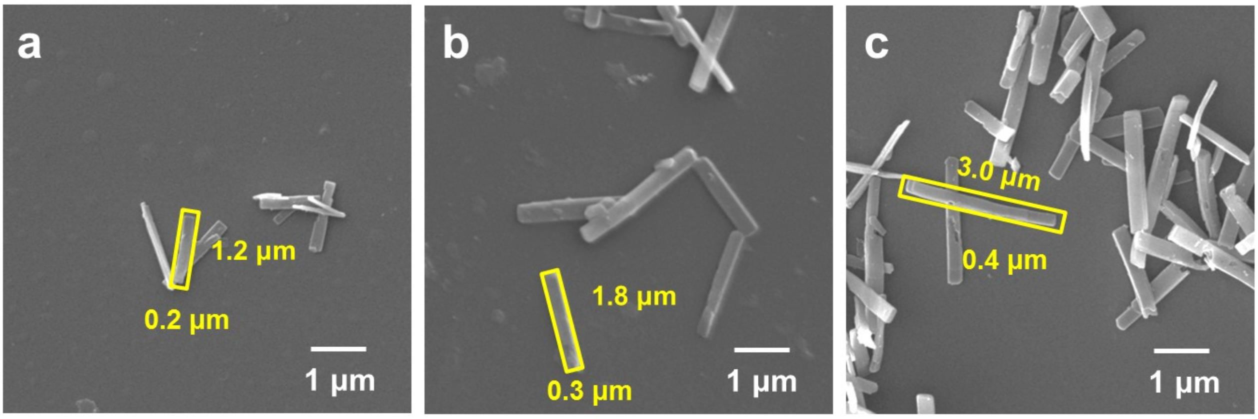

Supplementary Figure 42. SEM images of fresh SSP_{20} made from LSP_{seed} and SG7 solution with different concentrations: **(a)** 1.0 mM, **(b)** 2.5 mM and **(c)** 4.0 mM.

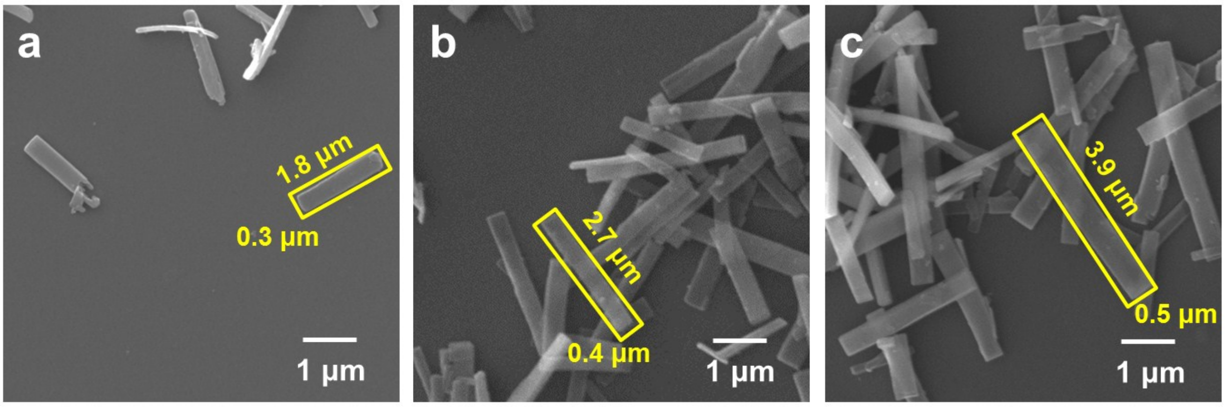

Supplementary Figure 43. SEM images of fresh SSP_{20} by different volume ratio of $SG7_{agg}/LSP_{seed}$: **(a)** 1:1, **(b)** 2:1 and **(c)** 4:1.

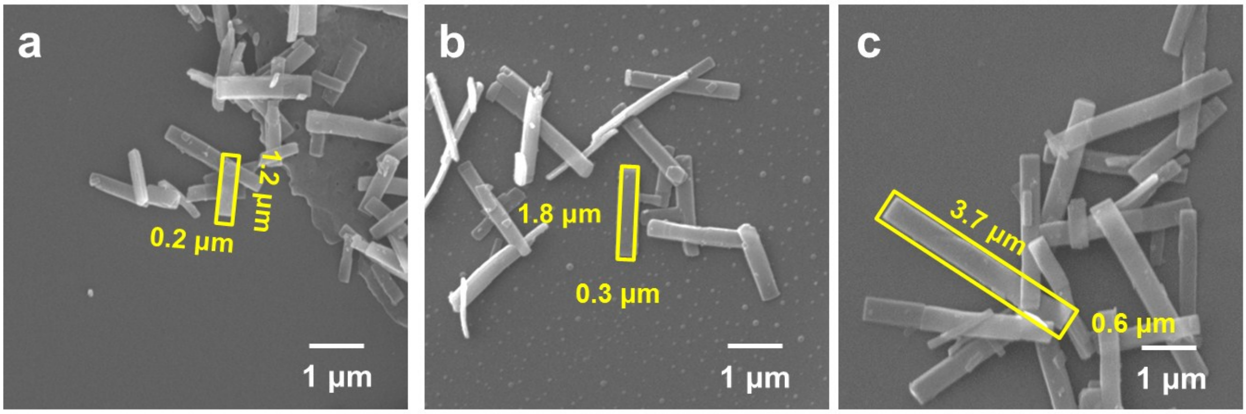

Supplementary Figure 44. SEM images of fresh SSP_{20} prepared with metastable LSP_{20} with different concentrations: **(a)** 0.25 mM, **(b)** 2.5 mM and **(c)** 5.0 mM.

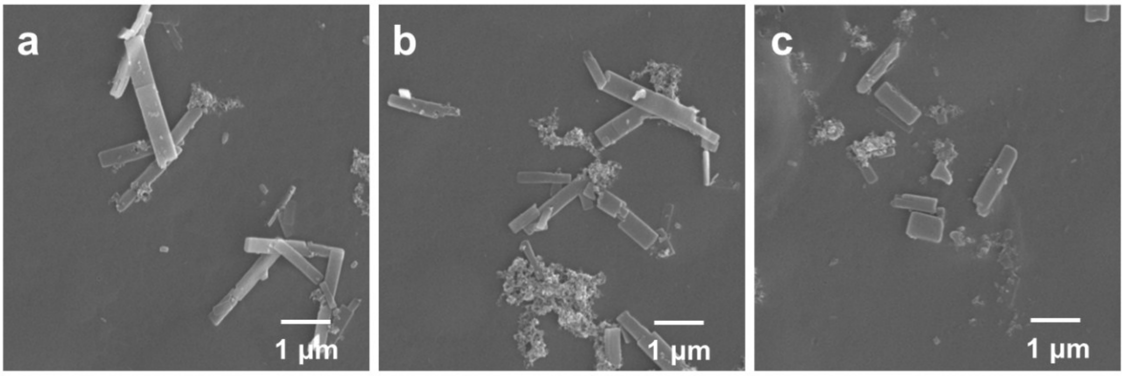

Supplementary Figure 45. SEM images of fresh **(a)** SSP_{50}, **(b)** SSP_{100} and **(c)** SSP_{3000} in Cycle 1.

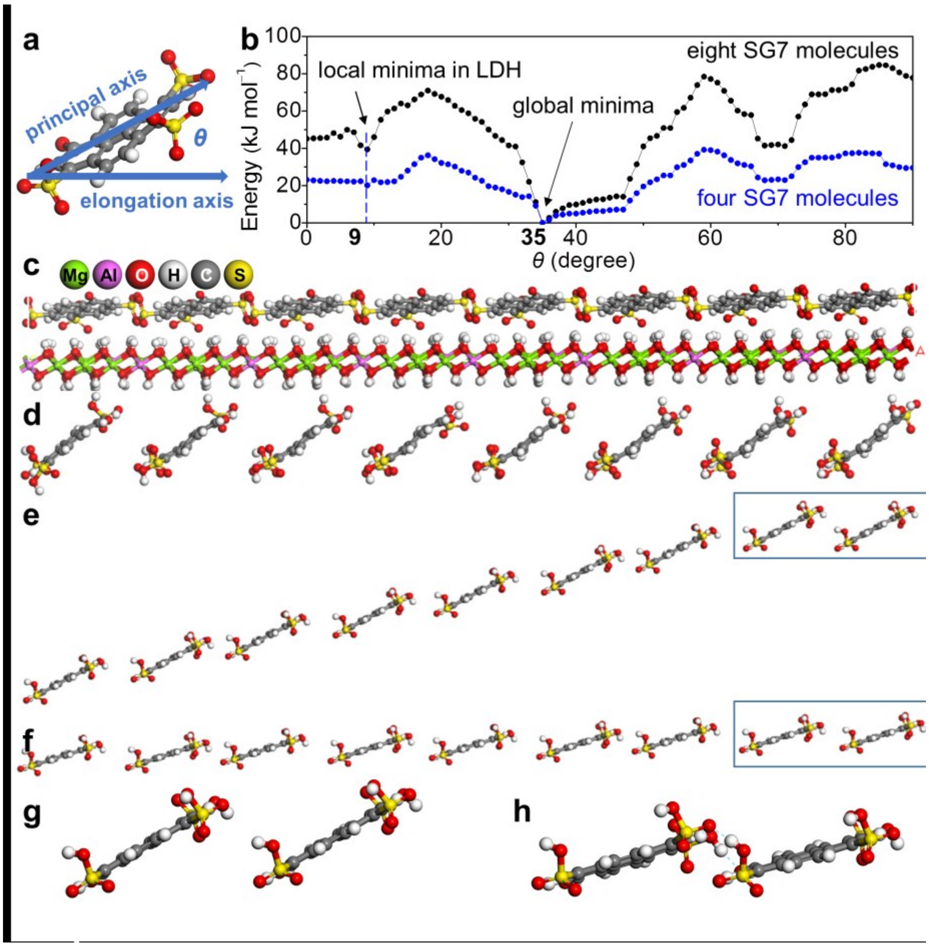

Supplementary Figure 46. (a) Schematic illustration for the definition of orientation angle, θ. **(b)** Energy diagram of SG7 LSM with different orientation. Optimized geometries of **(c)** SG7-LDH, **(d)** inactive SG7 aggregates (θ = 35º), **(e)** SG7 LSM+SG7 monomer (θ = 35º) and **(f)** SG7 LSM+SG7 monomer (θ = 9º), together with the highlighted binding areas in e and f: **(g)** θ = 35º and **(h)** θ = 9º. The color of each element is labeled in c.

The potential energy surface of $SG7_{agg}$ composed of eight SG7 molecules is completely searched with orientation angle (θ) of SG7 ranging from 0 to 90º with the step of 1º. The global energy minima is calculated to be at θ = 35º, which is deduced to be the geometry of thermodynamic equilibrium $SG7_{agg}$. The energy level of SG7 monomer is 167.0 kJ·mol^{-1} higher

than that of $SG7_{agg}$. The conversion from $SG7_{agg}$ (θ = 35º) to ordered SG7 in LDH (θ = 9º) needs to overcome an energy barrier of 70.55 kJ mol^{-1} at θ = 18º (the maximum of potential energy surface from 35º to 9º).

The effect of the number of SG7 molecules on the energy diagram is investigated. The potential energy surface of ordered SG7 composed of four molecules is also searched, as displayed in Supplementary Fig. 46b. The global energy minima also lies at θ = 35º, the same with that of ordered SG7 with eight molecules. The ordered SG7 in LDH, corresponding to θ = 9º, is also a local minima, in accordance with the ordered SG7 with eight molecules. For ordered SG7 with four molecules, the conversion of $SG7_{agg}$ (θ = 35º) to ordered SG7 in LDH (θ = 9º) needs to overcome an energy barrier of 36.22 kJ mol^{-1} at θ = 18º. In general, the energy barrier overcome by LDH confinement space is influenced by the number of SG7 molecules. But the locations of global minima and local minima are independent of the number of SG7 molecules.

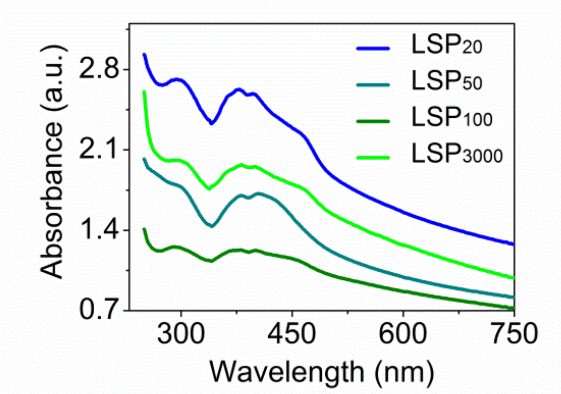

Supplementary Figure 47. UV-vis spectra of LSP_{20}–LSP_{3000} (optical path = 0.2 mm).

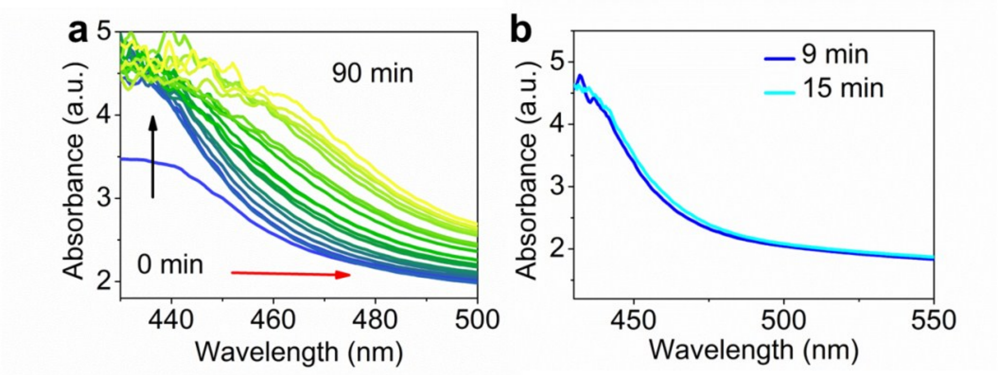

Supplementary Figure 48. Absorption spectra of metastable LSP_{20} without ultrasound at different time: **(a)** from 0 to 90 min and **(b)** at 9 and 15 min (optical path = 1 mm).

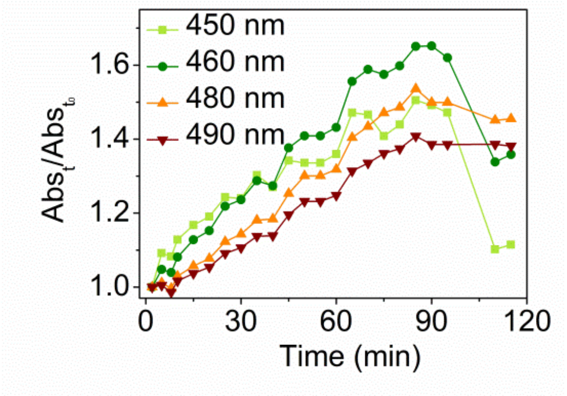

Supplementary Figure 49. Diagram of the ratio of time-dependent absorbance to initial value of metastable LSP_{20} without ultrasound at different wavelengths (optical path = 1 mm).

The FL spectra show that LSP_{20} without ultrasound has slower kinetics. Thus, FL spectra of LSP_{20} without ultrasound are used to observe more details. Although the quartz cuvette with the optical path of 0.2 mm can guarantee the absorbance in the instrument range (−5.5~5.5 Abs), it is unsuitable for the living polymerization of oversized LSP_{20}. Here, the cuvette with the optical path of 1 mm is chosen to detect the dynamics of LSP_{20}, which causes the absorbance to exceed the range of the photometer at ultraviolet region less than 400 nm. Therefore, the range of 450 nm to 490 nm is chosen to study the intensity change and wavelength shift in the UV-spectrum. At the initial stage of living polymerization (0−40 min), the absorbance intensity at the short wavelength (450 and 460 nm) increases rapidly. After 40 min, the absorbance at 450 nm shows large fluctuations and slower increasing rate, while the absorbance intensity at 460 nm shows faster increasing rate. In contrast, absorbance intensity at longer wavelength (both 480 and 490 nm) shows a relatively stable increase before 85 min, indicating that larger H-bond net forms during

living polymerization. In particular, after 90 min, the absorbance intensity decreases rapidly at 450 and 460 nm and tends to be flat at 480 and 490 nm, indicating that larger H-bond net may be a step-by-step process of developing from the smaller network.

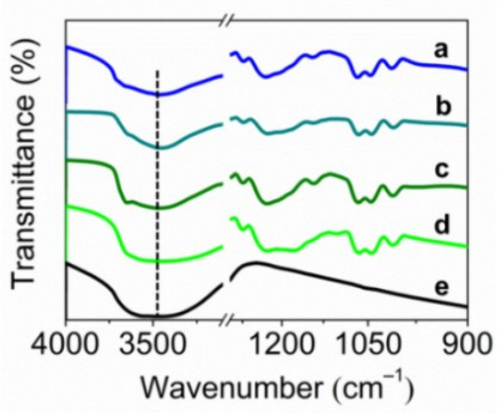

Supplementary Figure 50. FTIR spectra of SG7-LDH with the size of: (a) 20 nm, (b) 50 nm, (c) 100 nm and (d) 3 μm. (e) FTIR spectra of 20 nm LDH precursor.

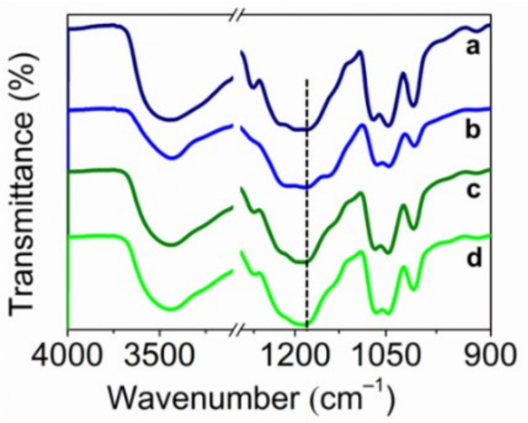

Supplementary Figure 51. FTIR spectra of (a) SG7 powder without further treatment and dried SG7 from different solvents: (b) CH_3OH/TFA (1:3 v/v), (c) H_2O and (d) CH_3OH.

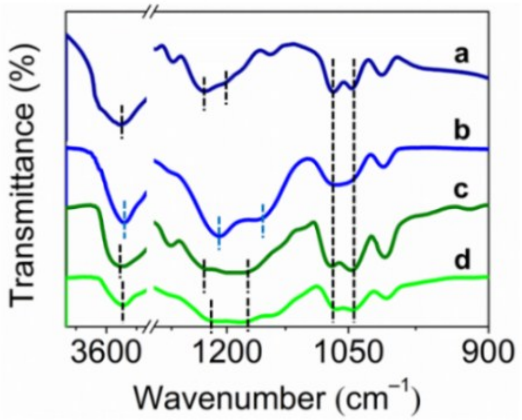

Supplementary Figure 52. FTIR spectra of (a) SG7-LDH, (b) LSP_{20}, (c) SG7 powder and (d) dried SG7 solution in CH_3OH/TFA (1:3 v/v).

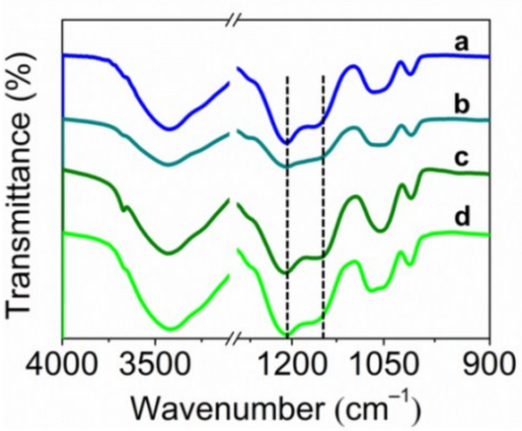

Supplementary Figure 53. FTIR spectra of (a) LSP_{20}, (b) LSP_{50}, (c) LSP_{100} and (d) LSP_{3000}.

The existence of H-bond net during living polymerization of LSP can also be explored by FTIR spectra (Supplementary Fig. 50–53). Firstly, some contrast samples are tested in FTIR spectra to exclude the influence of −OH in LDH and mixed solvents. In SG7-LDH, the wide peak of −OH is attributed to the interactions between $-SO_3^-$, −OH of SG7 and LDH precursor (3460 cm^{-1}) (Supplementary Fig. 50). After dissolving SG7 in CH_3OH/TFA (1:3 v/v), the −OH of SG7 shows a blue-shifted wavenumber (3432 cm^{-1}), compared with SG7 powder without further treatment (3450 cm^{-1}) (Supplementary Fig. 51). Finally, the −OH in LSP shifts to the lowest wavenumber (3420 cm^{-1}), attributing to larger H-bond net induced by confinement space in the same mixture solvent. Under normal experimental conditions, almost no change can be seen in the peak of antisymmetric stretching vibration (ν_{as}) and symmetric stretching vibration (ν_s) of $-SO_3^-$ in dried SG7 powder with any other treatment (Supplementary Fig. 52). Among all samples, only LSP shows distinctive peak shape of $-SO_3^-$. The ν_{as} of $-SO_3^-$ in supramolecular polymers shows two split peaks at 1209 and 1151 cm^{-1} and ν_s of $-SO_3^-$ becomes weaker than ν_{as} (Supplementary Fig. 52–53). Herein, for O atom of $-SO_3^-$ in antisymmetric environment, O atoms are involved in the hydrogen bond with −OH, leading to the split of $\nu_{as}(-SO_3^-)$. The larger $\nu_{as}(-SO_3^-)$ splits, the

stronger the coulomb force or hydrogen bond is. Meanwhile, SG7-LDH and SG7 powder show unclear split of $\nu_{as}(-SO_3^-)$ at 1186 cm^{-1} and stronger peak of $\nu_s(-SO_3^-)$ at 1045 cm^{-1}, indicating that the hydrogen bond can be hardly tested.

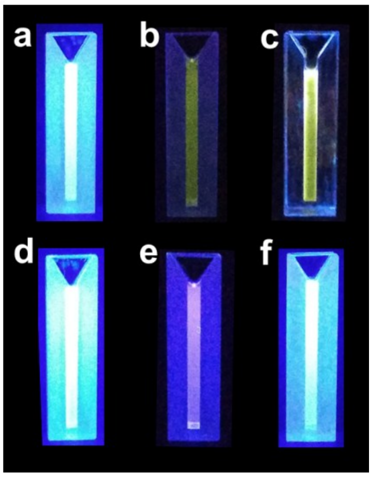

Supplementary Figure 54. The effect of H-bond on the stability of LSP. LSP dispersed in **(a)** CH_3OH, **(b)** CH_3CN and **(c)** CH_3OH/TFA (1:3 v/v). **(d−f)** SG7 powder dissolved in the same solvents as a−c, respectively.

As can be seen, the color of LSP acetonitrile (CH_3CN) solution undergoes no change; while the color of LSP in ethanol (CH_3OH) solution instantly changes from yellow to blue-light, indicating that LSP depolymerizes into SG7 monomer because the H-bond net is replaced by the H-bond between solvent CH_3OH and SG7.

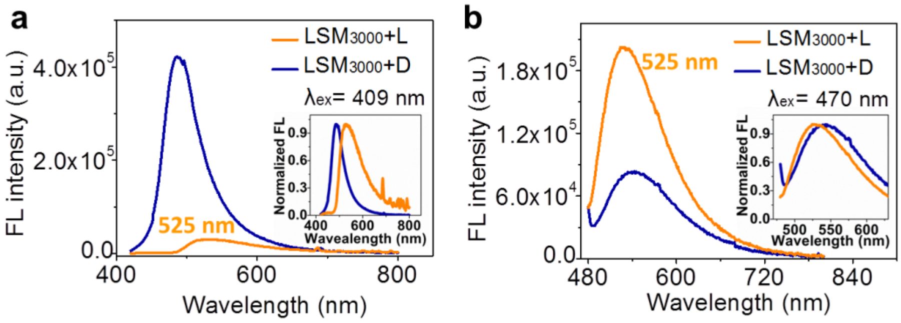

Supplementary Figure 55. FL emission spectra of chiral recognition products of LSM_{3000}+L and LSM_{3000}+D after ultrasound for 50 min (insets: corresponding normalized spectra).

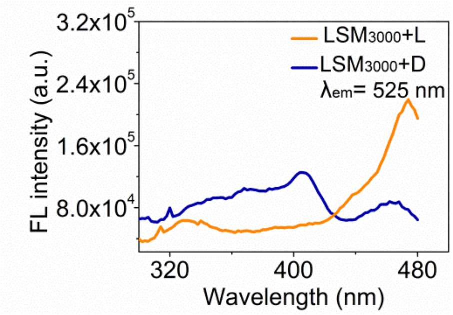

Supplementary Figure 56. FL excitation spectra of chiral recognition products of LSM_{3000}+L and LSM_{3000}+D.

For the polymer-L_{3000}, only the peak for J-agg fragment appears at 525 nm. The blue-shift of emission (λ_{em} = 525 nm, Supplementary Fig. 55) of polymer-L_{3000} can be observed compared with normally elongated LSP_{3000} (λ_{em} = 535 nm, 4.00 μm^2, Supplementary Fig. 11 and Fig. 2h). These phenomena are indirect evidences of that L-Arg participates in the assembly of polymer-L_{3000} and plays an isolating role between LSM_{3000}, reducing the long-range array and the size of following supramolecular polymers. However, at same ultrasound time with 50 min, the mixture solution shows a LSM_{3000} emission at 485 nm (λ_{ex} = 409 nm) and a normally elongated LSP_{3000} emission at 535 nm (λ_{ex} = 470 nm), indicating that polymer-D_{3000} still fails to appear. This difference in the type of polymerization caused by adding different L- or D-Arg can also be confirmed by the excitation spectrum (Supplementary Fig. 56).

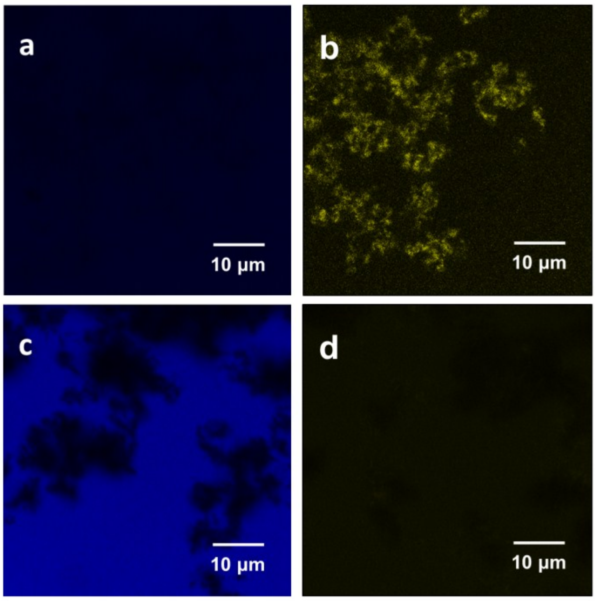

Supplementary Figure 57. CLSM images of formed polymer-L_{3000} at 50 min by the co-assembly of LSM_{3000} and L-Arg: **(a)** $\lambda_{\text{laser diode}}$ = 405 nm, **(b)** $\lambda_{\text{laser diode}}$ = 488 nm. CLSM images of chiral products of LSP_{3000}+D at 50 min: **(c)** $\lambda_{\text{laser diode}}$ = 405 nm and **(c)** $\lambda_{\text{laser diode}}$ = 488 nm.

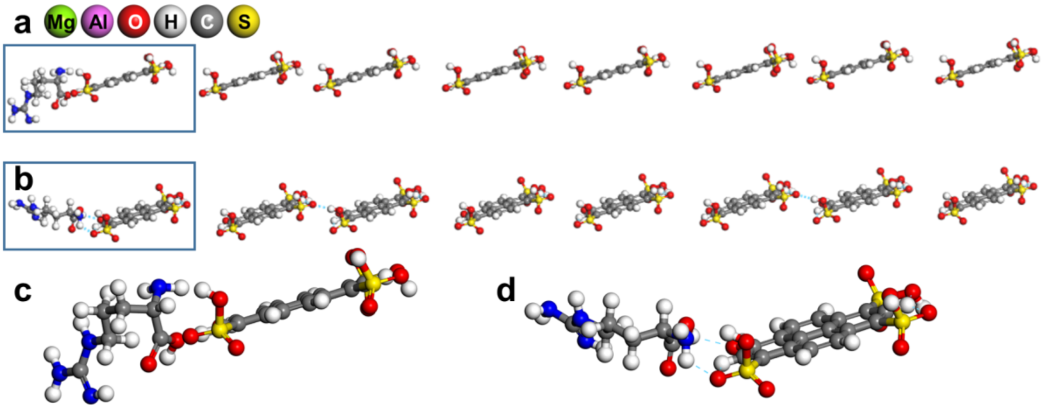

Supplementary Figure 58. Optimized geometries of **(a)** LSM_{3000}+L, **(b)** LSM_{3000}+D, **(c)** highlighted binding area in a, and **(d)** highlighted binding area in b.

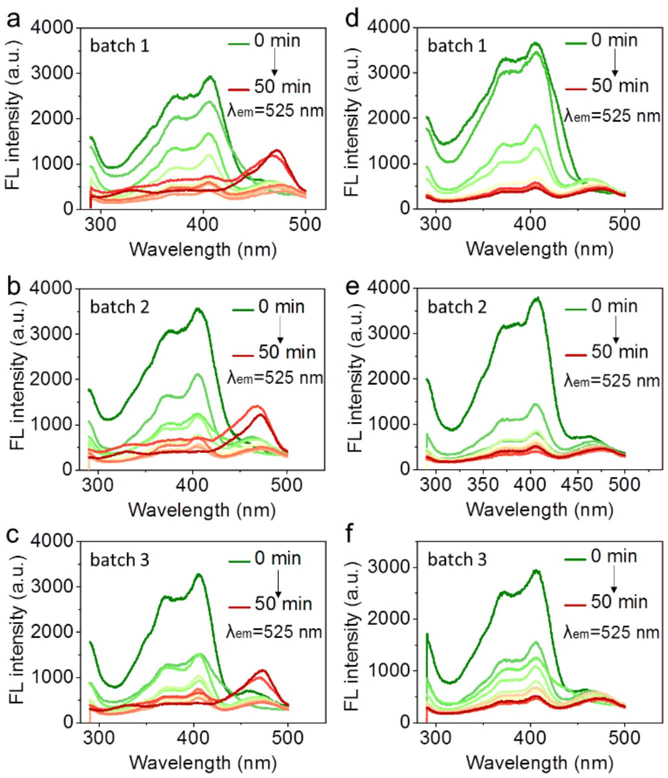

Supplementary Figure 59. FL excitation spectra of chiral recognition products with different ultrasound time: **(a−c)** LSM_{3000}+L in batch 1−3 and **(d−f)** LSM_{3000}+D in batch 1−3.

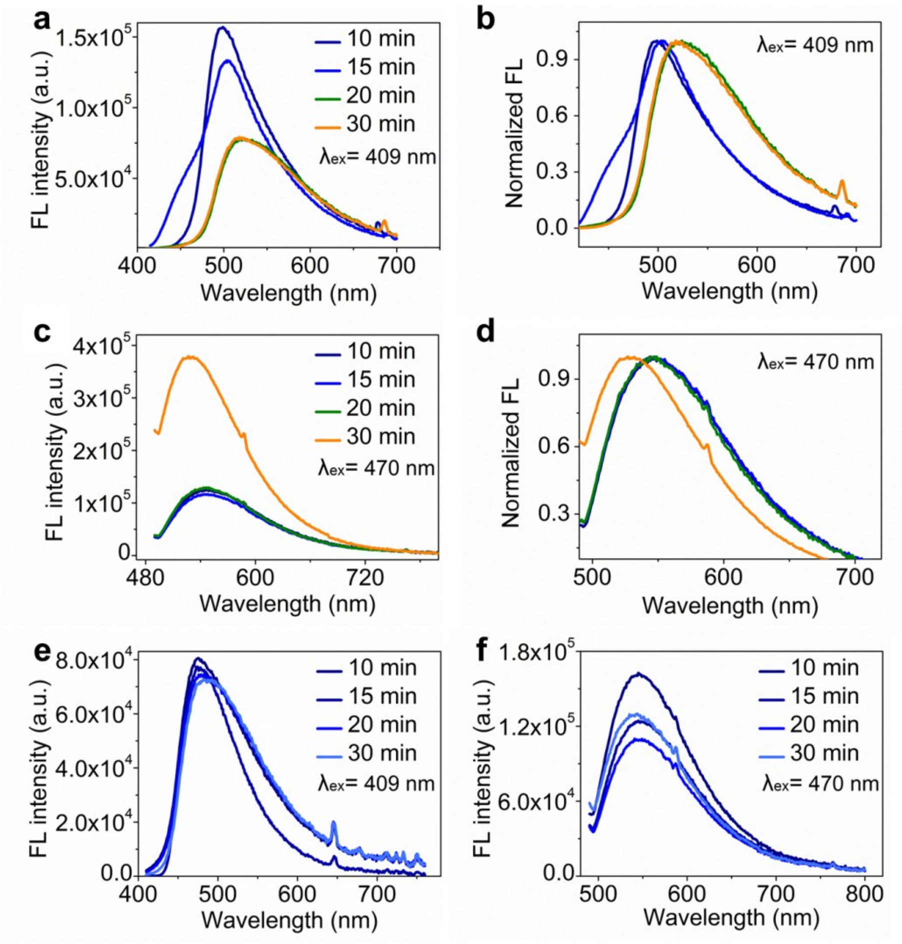

Supplementary Figure 60. FL emission spectra of chiral recognition products of **(a−d)** LSM_{100}+L and **(e−f)** LSM_{100}+D with different ultrasound time, b and d are corresponding normalized spectra in a and c, respectively. Noted that the effective recognition is within 20 min after end of ultrasound.

At same time with 20 min, the mixture solution of LSM_{100} and L- or D-Arg shows the absence of LSM_{100} emission at 480 nm (λ_{ex} = 409 nm) and appearance of the normally elongated LSP_{100} emission at 535 nm (λ_{ex} = 470 nm), indicating that polymer-L_{100} still fails to appear

(Supplementary Fig. 60a–d). Until 30 min, the formation of polymer-L_{100} can be observed by appearance of the emission peak at 525 nm (Supplementary Fig. 60c–d), which shows a blue-shift compared with normally elongated LSP_{100} (λ_{em} = 535 nm, Supplementary Fig. 11). The lag time between the disappearance of LSM and the formation of polymer-L_{100} indicates a transition state made of L-Arg and LSM may exist before formed polymer-L_{100}. Slower kinetics of co-assembly on LSM_{100} and D-Arg provides possibilities for chiral recognition, which proved by large amounts of existing LSM_{100} ($\lambda_{em} \approx$ 480 nm, Supplementary Fig. 60e) and normally elongated LSP_{100} (λ_{em} = 535 nm, Supplementary Fig. 60f) after ultrasound for 30 min.

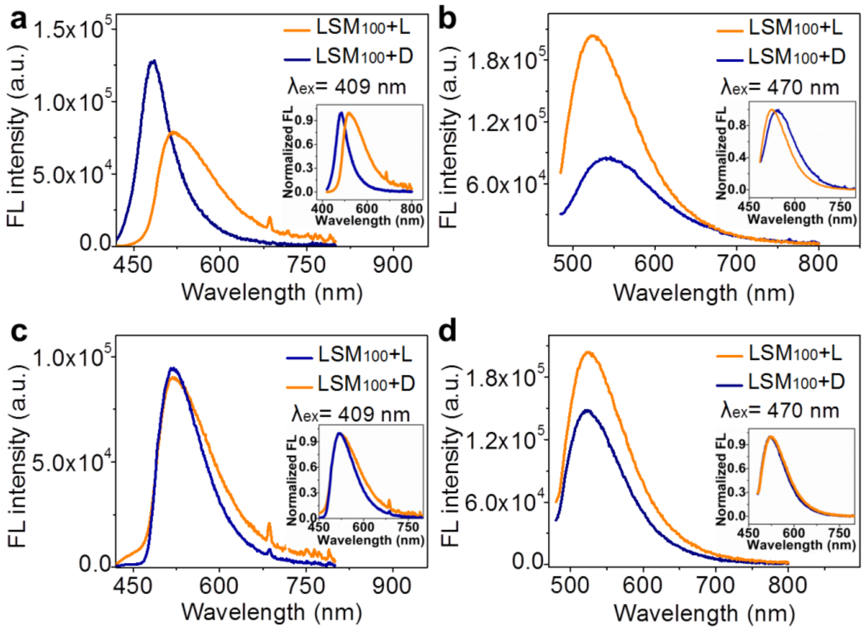

Supplementary Figure 61. FL emission spectra of chiral recognition products of LSM_{100}+L and LSM_{100}+D **(a–b)** upon end of ultrasound and **(c–d)** after kept for 20 min. (insets: corresponding normalized spectra).

Unlike the metastable LSM_{3000}, recognition of metastable LSM_{100} to L- or D-Arg can be maintained in 20 min when ultrasound just stopped. In other words, the lag time of polymer-D_{100} and polymer-L_{100} is different by 20 min. Before 20 min, the emission peak of polymer-L_{100} is at 523 nm excited by either 409 nm or 470 nm, but the mixture of metastable LSM_{100} and D-Arg possesses the blue light of metastable LSM_{100} at 480 nm and yellow light of elongated LSP_{100} at 535 nm. After 20 min, both polymer-L_{100} and polymer-D_{100} are generated, displaying the same emission spectrum. Polymer-L_{100} and polymer-D_{100} cannot be recognized by naked eyes.

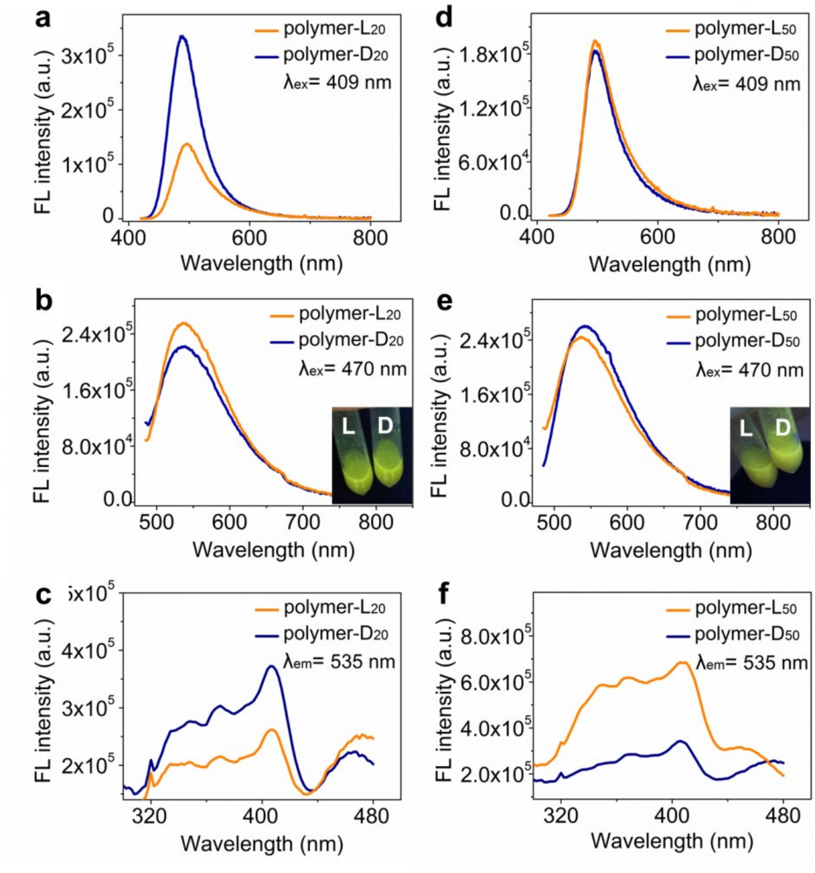

Supplementary Figure 62. Invalid chiral recognition of metastable LSM_{20} and LSM_{50} to L- or D-Arg. **(a−b)** FL emission spectra and **(c)** excitation spectra of polymer-L_{20} and polymer-D_{20}. **(d−e)** FL emission spectra and **(f)** excitation spectra of polymer-L_{50} and polymer-D_{50}.

After 10 min of ultrasound, polymer-D_{20} and polymer-L_{20} are generated almost at the same time. Neither emission nor excitation spectra can achieve effective recognition. The same result also happens to the metastable LSM_{50}. Moreover, even though polymer-L_{20}, polymer-D_{20}, polymer-L_{50} and polymer-D_{50} have been generated with yellow light at ~535 nm, there still exists the blue light of LSM at ~490 nm, indicating that there is a subtle dynamics balance between co-assembly (Arg and LSM) and self-assembly of metastable LSM.

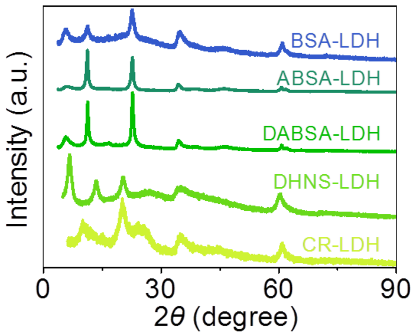

Supplementary Figure 63. XRD patterns of BSA-LDH, ABSA-LDH, DABSA-LDH, DHNS-LDH and CR-LDH.

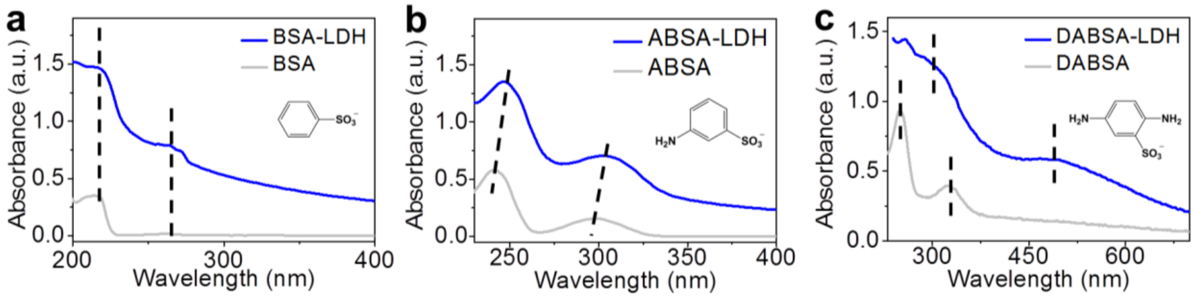

Supplementary Figure 64. UV-vis spectra of intercalated LDH (20 g/L in CH_3OH) and corresponding contrast samples (5 mM in CH_3OH) (optical path = 0.1 mm): **(a)** BSA-LDH, **(b)** ABSA-LDH and **(c)** DABSA-LDH.

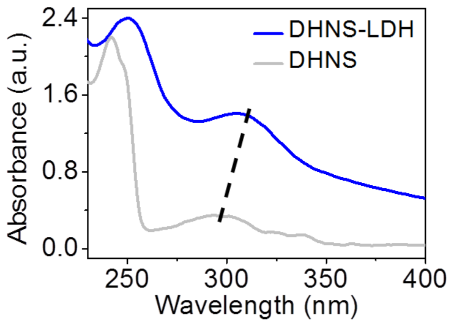

Supplementary Figure 65. UV-vis spectra of DHNS-LDH (10 g/L) and DHNS (5 mM) in CH_3OH (optical path = 0.1 mm).

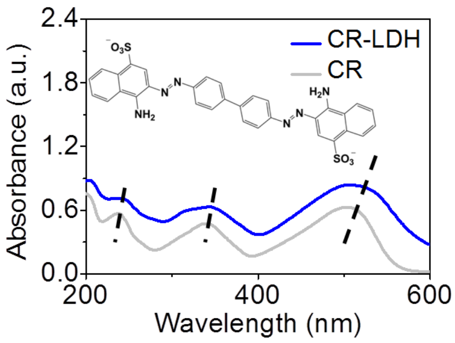

Supplementary Figure 66. UV-vis spectra of CR-LDH (2 g/L in CH_3OH) and corresponding contrast samples (1 mM in CH_3OH) (optical path = 0.1 mm).

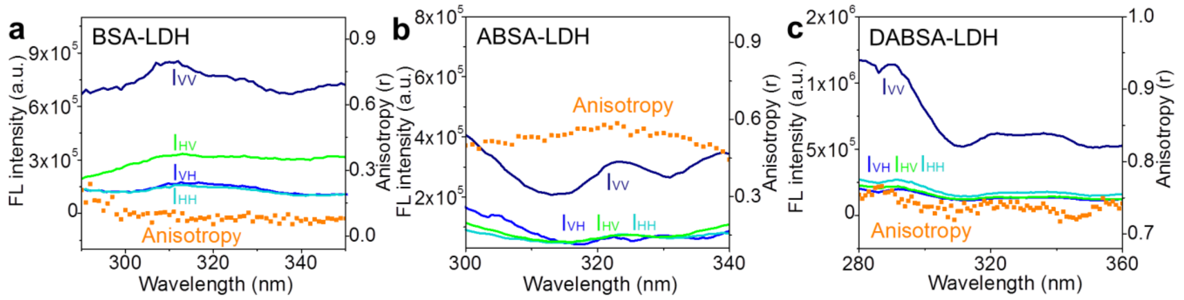

Supplementary Figure 67. Polarized FL profiles and anisotropic value (*r*) for **(a)** BSA-LDH (r = 0.104), **(b)** ABSA-LDH (r = 0.586) and **(c)** DABSA-LDH (r = 0.760) in solid state on the quartz plate, respectively.

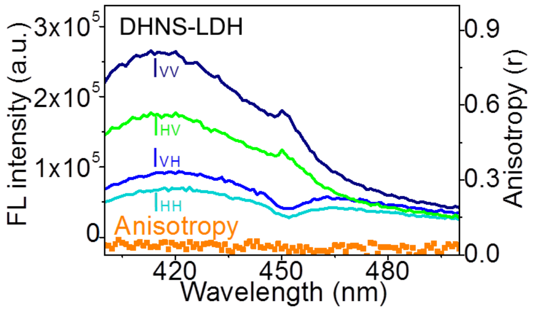

Supplementary Figure 68. Polarized FL profiles and anisotropic value (*r*) for DHNS-LDH (*r* = 0.0214) in solid state on the quartz plate.

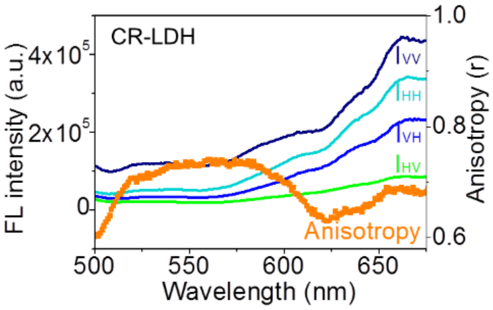

Supplementary Figure 69. Polarized FL profiles and anisotropic value (*r*) for CR-LDH (*r* = 0.691) in solid state on the quartz plate.

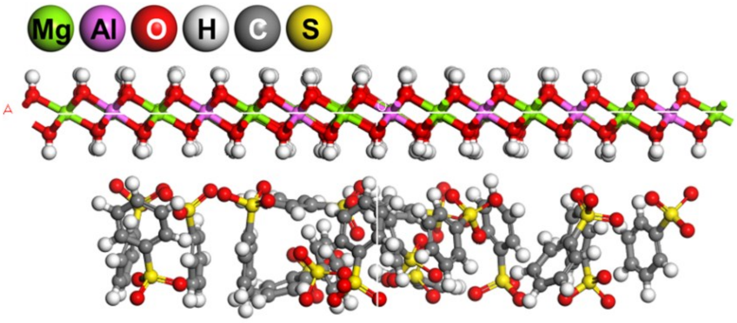

Supplementary Figure 70. The snapshot of BSA-LDH after AIMD simulations of 100 ps.

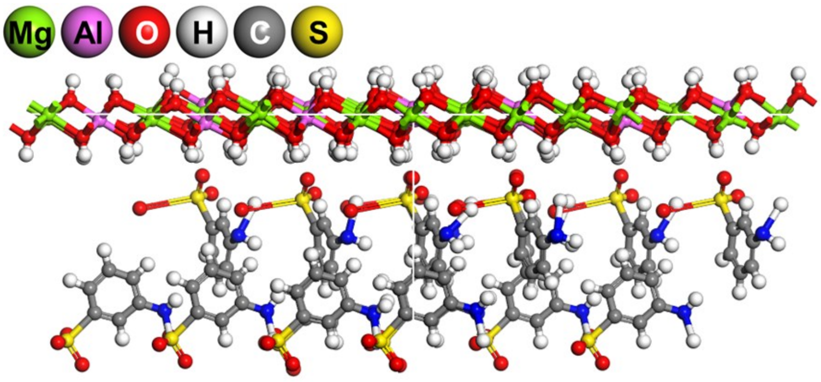

Supplementary Figure 71. The snapshot of ABSA-LDH after AIMD simulations of 100 ps.

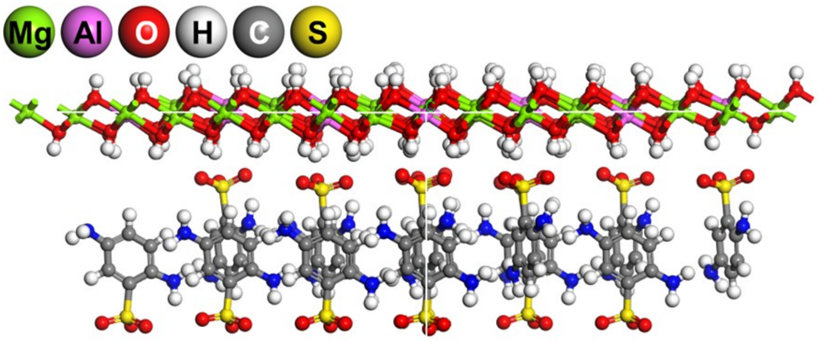

Supplementary Figure 72. The snapshot of DABSA-LDH after AIMD simulations of 100 ps.

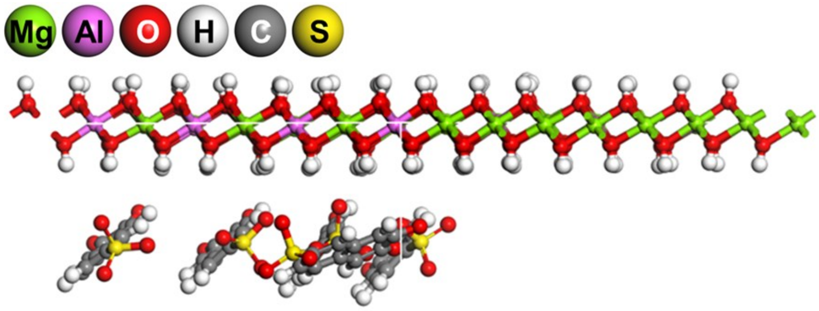

Supplementary Figure 73. The snapshot of DHNS-LDH after AIMD simulations of 100 ps.

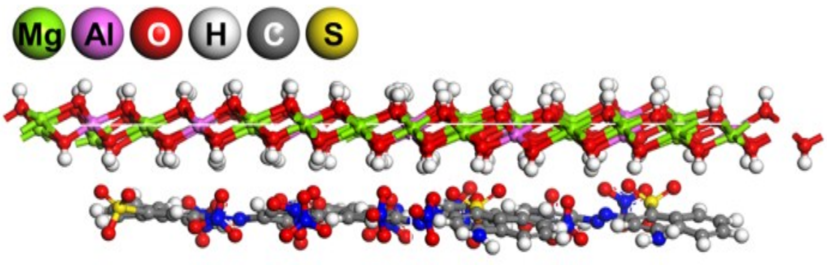

Supplementary Figure 74. The snapshot of CR-LDH after AIMD simulations of 100 ps.

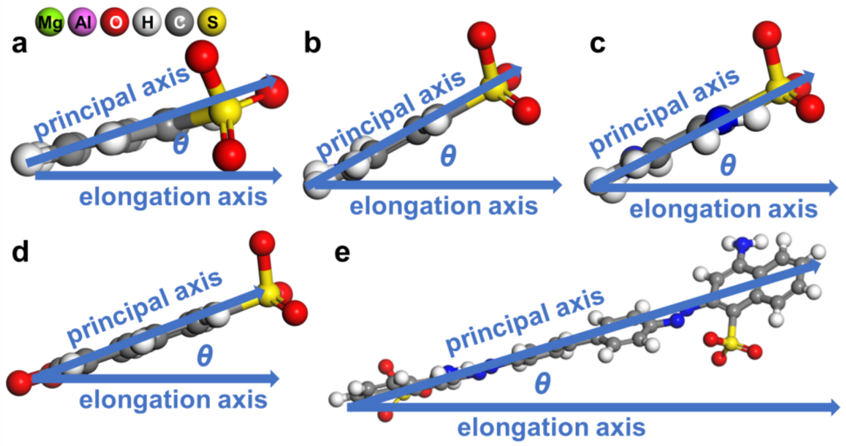

Supplementary Figure 75. Schematic illustration for the definition of θ for **(a)** BSA, **(b)** ABSA, **(c)** DABSA, **(d)** DHNS, and **(e)** CR.

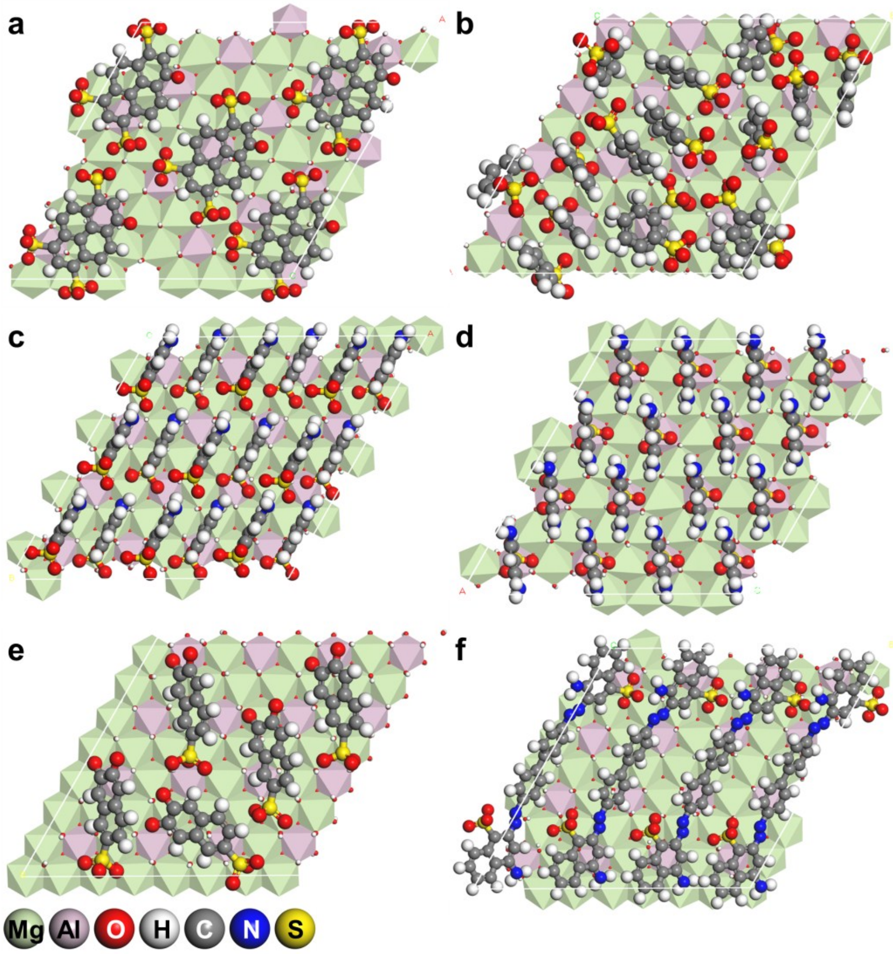

Supplementary Figure 76. The top view of intercalated LDHs: **(a)** SG7-LDH, **(b)** BSA-LDH, **(c)** ABSA-LDH, **(d)** DABSA-LDH, **(e)** DHNS-LDH and **(f)** CR-LDH.

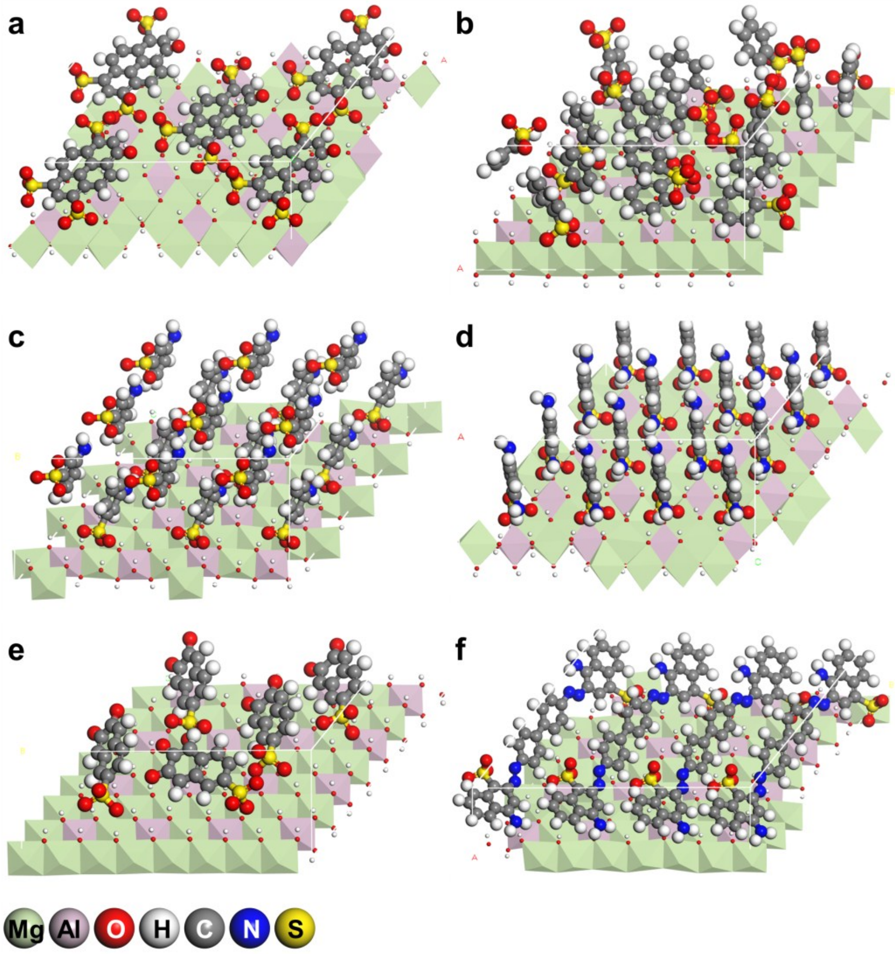

Supplementary Figure 77. The side view of intercalated LDHs: **(a)** SG7-LDH, **(b)** BSA-LDH, **(c)** ABSA-LDH, **(d)** DABSA-LDH, **(e)** DHNS-LDH and **(f)** CR-LDH.

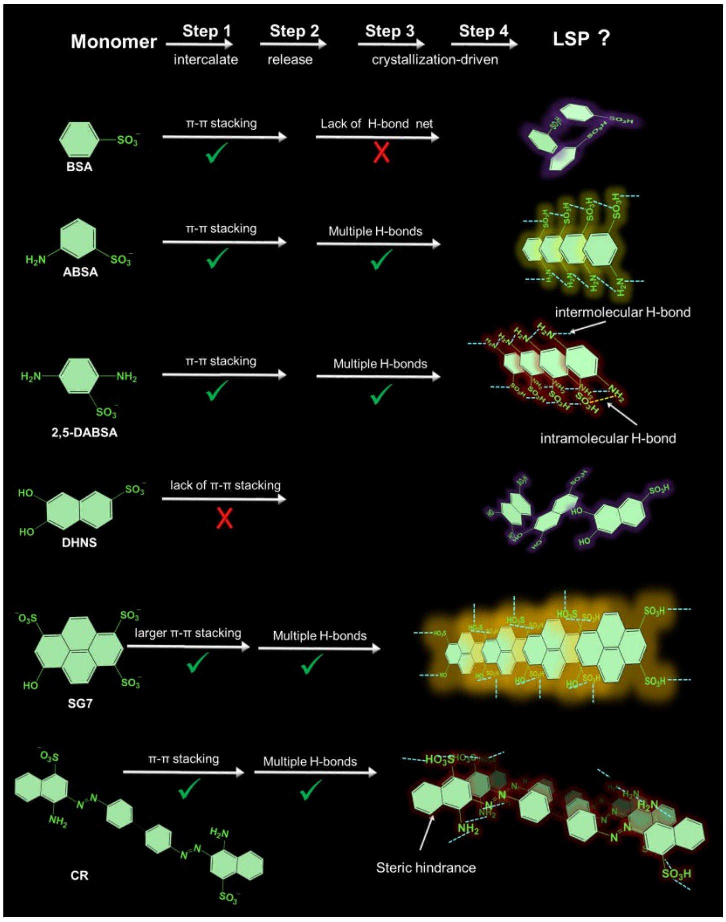

Supplementary Figure 78. Schematic illustration for various simple-structured molecules going through steps 1−4 in our proposed method and applicable molecular characteristics. The molecule structures are observed from ChemDraw and adapted to various assemblies by author in Microsoft Office PowerPoint.

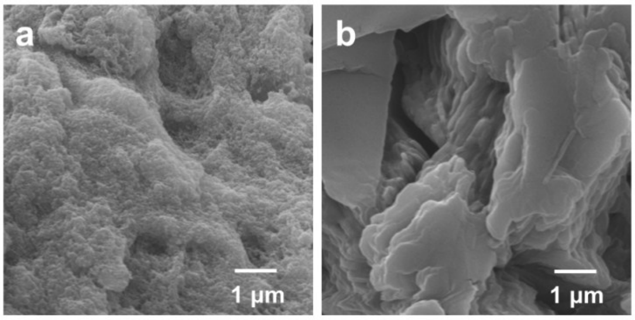

Supplementary Figure 79. SEM images of **(a)** failed LSP made of BSA and **(b)** corresponding contrast sample: physically mixing BSA and Cl-LDH in CH_3OH/TFA (1:3 v/v) with the same dosage as LSP.

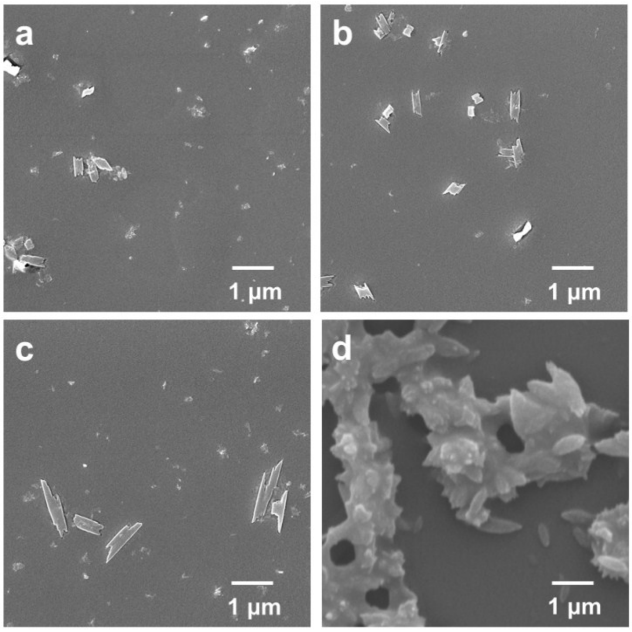

Supplementary Figure 80. SEM images of **(a)** fresh metastable LSP made of 3-ABSA, corresponding fresh SSPs in **(b)** Cycle 1 and **(c)** Cycle 2, and **(d)** contrast sample: physically mixing pure 3-ABSA and Cl-LDH in CH_3OH/TFA (1:3 v/v) with the same dosage as LSP.

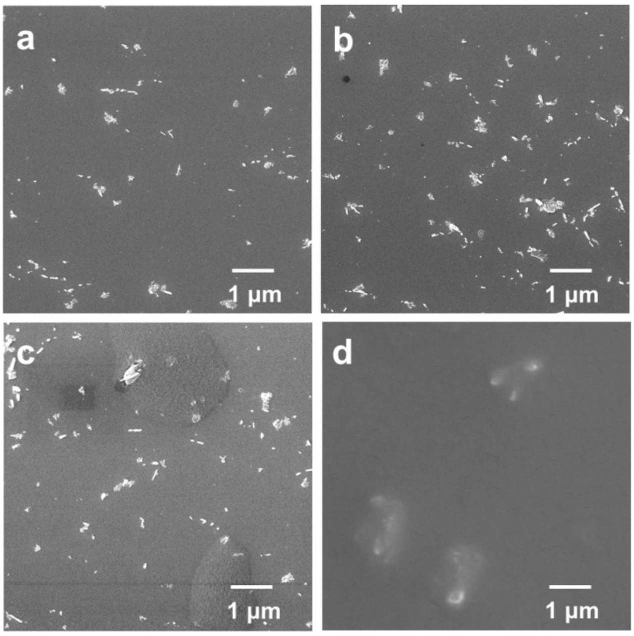

Supplementary Figure 81. SEM images of **(a)** fresh metastable LSP made of 2,5-DABSA, corresponding fresh SSPs in **(b)** Cycle 1 and **(c)** Cycle 2, and **(d)** contrast sample: physically mixing pure 2,5-DABSA and Cl-LDH in CH_3OH/TFA (1:3 v/v) with the same dosage as LSP.

Besides, compared with 3-ABSA, the $-SO_3H$ of 2,5-DABSA tends to form intramolecular H-bond with the adjacent $–NH_2$, theoretically avoiding the risk of spontaneous nucleation to a greater extent. However, the size of LSP made of 2,5-DABSA (Supplementary Fig. 81) is much smaller than 3-ABSA (Supplementary Fig. 80), indicating that intramolecular H-bond is unfavorable to the formation process of LSP.

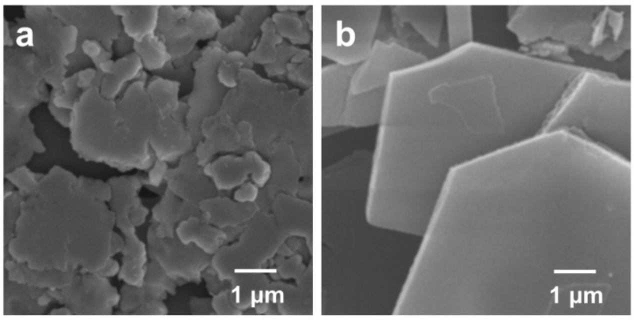

Supplementary Figure 82. SEM images of **(a)** failed LSP made of DHNS and **(b)** contrast sample: physically mixing DHNS and Cl-LDH in CH_3OH/TFA (1:3 v/v) with the same dosage as LSP.

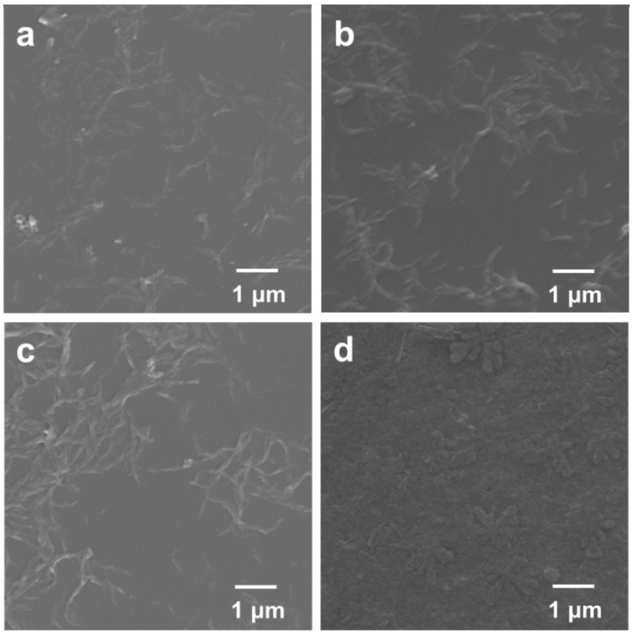

Supplementary Figure 83. SEM images of **(a)** fresh metastable LSP made of CR, corresponding fresh SSPs in **(b)** Cycle 1 and **(c)** Cycle 2, and **(d)** contrast sample: physically mixing pure CR and Cl-LDH in CH_3OH/TFA (1:3 v/v) with the same dosage as LSP.

The introduction of steric hindrance is a double-edged sword, which not only inhibits spontaneous nucleation but also adversely affects the elongation of LSP. Compared with 3-ABSA and 2,5-DABSA, CR shows a large steric hindrance on both sides. However, the size of LSP made of CR is the smallest (Supplementary Fig. 83), indicating the limitation of steric hindrance in the preparation of large LSP.

3. Supplementary Tables

Supplementary Table 1. The ICP-MS results of pure Cl-LDH precursors with different sizes in our work, the calculated ratio of Mg/Al and corresponding chemical formula

Various LDHs	Mg (ppm)	Al (ppm)	Mg/Al	$[Mg_aAl_b(OH)_x](A^{n-})_y{\cdot}zH_2O$	
				a	*b*
LDH_{20}	3.89	1.41	3.06	0.75	0.25
LDH_{50}	5.02	1.77	3.15	0.76	0.24
LDH_{100}	6.49	2.44	2.95	0.75	0.25
LDH_{3000}	4.19	1.65	2.82	0.74	0.26

Supplementary Table 2. Elemental analysis results of SG7-LDH and SG7-LDH_{20}-surface in our work and the calculated weight fraction of SG7 and corresponding chemical formula

Various LDHs	S (wt.%)	SG7(wt.%)	average of SG7 (wt.%)
SG7-LDH_{20}-1	5.47	25.93	
SG7-LDH_{20}-2	5.74	27.21	26.32
SG7-LDH_{20}-3	5.45	25.83	
SG7-LDH_{20}-surface-1	0.66	3.13	
SG7-LDH_{20}-surface-2	0.66	3.13	3.11
SG7-LDH_{20}-surface-3	0.65	3.08	

In SG7-LDH_{20} system, SG7 accounts for 26.32 wt.%. Among them, the adsorption capacity of SG7 on the outer surface of CO_3-LDH (named as SG7-LDH_{20}-surface) is 3.11 wt.%; the intercalation capacity of SG7 in interlayer of LDH is 23.21 wt.%. That is, the adsorption capacity only accounts for 13.41% of the total SG7.

Supplementary Table 3. The number-average area (A_n), weight-average area (A_w), and PDI (A_w/A_n) of metastable LSP_{20}–LSP_{3000}, respectively, which was obtained by evaluating over 50 objects in SEM images

Various LSPs	A_n (μm^2)	A_w (μm^2)	PDI (A_w/A_n)
LSP_{20}	0.03880	0.04035	1.040
LSP_{50}	0.07115	0.07657	1.076
LSP_{100}	0.1131	0.1192	1.054
LSP_{3000}	0.7639	0.8144	1.066

Supplementary Table 4. Parameters in Debye plot of $Kc/R_\theta P_\theta$ (mol kg^{-1}) and metastable LSP concentration

**LDH precursor**	**Intercept $(mol·kg^{-1})×10^{-3}$**	**slope(mol·L·kg^{-2})**	R^2	**DP**
20 nm	46.4	9.82	0.929	41.10
50 nm	4.36	0.287	0.985	437.4
100 nm	0.476	2.51	0.999	4006
3 μm	0.289	0.604	0.998	6598

Here, corrected scattering intensity of sample is measured to calculate Kc/R_θ, and then Debye plot can be obtained according to Rayleigh equation (Equation (2)):

$$\frac{Kc}{R_\theta} = \left(\frac{1}{M}+2A_2c\right)P_\theta \qquad (2)$$

where K is optical constant, c is sample concentration (g L^{-1}), R_θ is Rayleigh ratio and toluene acts as a reference, M is M_w of sample, A_2 is second virial coefficient and P_θ is the particle scattering function.

Supplementary Table 5. Parameters in linear relationship of corrected scattering (kcps) and metastable LSP concentration

**LDH precursor**	**Intercept (kcps)×10^{-3}**	**slope**	$\boldsymbol{R^2}$
20 nm	3.90	0.290	0.975
50 nm	1.98	2.03	0.995
100 nm	7.69	0.361	0.976
3 μm	4.79	1.48	0.997

Supplementary Table 6. Comparison of average size and DP between previous work and metastable LSP_{3000}

monomer	Average length	Average width	DP	Ref.
porphyrin derivatives	~250 nm			8
1,3-benzenedithiol	745 nm			9
porphyrin derivatives	~600 nm	~250 nm		10
perylene diimide derivatives	~500 nm	~60 nm		11
corannulene derivatives	~1 µm		900	12
platinum(II) complexes	~1 µm	~12 nm		13
porphyrin derivatives	~700 nm			14
[c2]daisy chain rotaxanes			2937±290	15
C3-symmetrical molecules			>1000	16
SG7	~900 nm	~200 nm	6598	Our work

Supplementary Table 7. The elemental analysis results of all intercalated LDHs in our work and the calculated weight fraction of intercalated molecules and corresponding chemical formula.

Various LDHs	S (wt.%)	O (wt.%)	H (wt.%)	A^{n-} (wt.%)	$[Mg_aAl_b(OH)_x](A^{n-})_y{\cdot}zH_2O$		
					x	*y*	*z*
SG7-LDH_{20}	5.47	33.40	4.73	25.93	1.93	0.08	2.2
SG7-LDH_{50}	5.72	33.90	4.40	27.11	1.80	0.11	2.9
SG7-LDH_{100}	5.69	33.61	4.54	26.97	1.85	0.10	2.5
SG7-LDH_{3000}	5.70	33.63	4.60	27.02	1.90	0.09	2.4
BSA-LDH_{20}	5.07	32.01	5.45	25.03	2.03	0.22	2.1
ABSA-LDH_{20}	5.21	29.94	5.11	28.17	1.98	0.27	2.3
DABSA-LDH_{20}	4.90	29.34	4.98	28.79	1.96	0.29	2.6
DHNS-LDH_{20}	3.32	24.05	3.94	24.80	2.04	0.08	2.5
CR-LDH_{20}	3.54	29.19	5.22	35.95	1.80	0.10	2.7

In detail, according to the results of ICP and elemental analysis, for the SG7-LDH_{20}, SG7-LDH_{50}, SG7-LDH_{100}, SG7-LDH_{3000}, BSA-LDH_{20}, ABSA-LDH_{20}, DABSA-LDH_{20}, DHNS-LDH_{20} and CR-LDH_{20}, the chemical formula are calculated to be $[Mg_{0.75}Al_{0.25}(OH)_{1.93}](SG7)_{0.08}{\cdot}2.2H_2O$, $[Mg_{0.76}Al_{0.24}(OH)_{1.80}](SG7)_{0.11}{\cdot}2.9H_2O$, $[Mg_{0.75}Al_{0.25}(OH)_{1.85}](SG7)_{0.10}{\cdot}2.5H_2O$, $[Mg_{0.74}Al_{0.26}(OH)_{1.90}](SG7)_{0.09}{\cdot}2.4H_2O$, $[Mg_{0.75}Al_{0.25}(OH)_{2.03}](BSA)_{0.22}{\cdot}2.1H_2O$, $[Mg_{0.75}Al_{0.25}(OH)_{1.98}](ABSA)_{0.27}{\cdot}2.3H_2O$, $[Mg_{0.75}Al_{0.25}(OH)_{1.96}](DABSA)_{0.29}{\cdot}2.6H_2O$, $[Mg_{0.75}Al_{0.25}(OH)_{2.04}](DHNS)_{0.08}{\cdot}2.5H_2O$ and $[Mg_{0.75}Al_{0.25}(OH)_{1.80}](CR)_{0.1}\ Cl_{0.25}{\cdot}2.7H_2O$, respectively.

4. Supplementary References

[1] Delley, B. An all-electron numerical method for solving the local density functional for polyatomic molecules. *J. Chem. Phys.* **92,** 508−517 (1990).

[2] Delley, B. From molecules to solids with the DMol^3 approach. *J. Chem. Phys.* **113,** 7756 (2000).

[3] Perdew, J. P., Burke, K. & Ernzerhof, M. Generalized gradient approximation made simple. *Phys. Rev. Lett.* **77,** 3865−3868 (1996).

[4] Tkatchenko, A. & Scheffler, M. Accurate molecular van der Waals interactions from ground-state electron density and free-atom reference data. *Phys. Rev. Lett.* **102,** 073005 (2009).

[5] Andersen, H. C. Molecular dynamics simulations at constant pressure and/or temperature. *J. Chem. Phys.* **72**, 2384-2393 (1980).

[6] Berendsen, H. J. C., Postma, J. P. M., Van Gunsteren, W. F., DiNola, A., & Haak, J. R. Molecular dynamics with coupling to an external bath. *J. Chem. Phys.* **81**, 3684-3690 (1984).

[7] Fu, H.-R., Wu, X.-X. Ma, L.-F., Wang, F. & Zhang, J. Dual-emission SG7@MOF sensor via SC−SC transformation: Enhancing the formation of excimer emission and the range and sensitivity of detection. *ACS Appl. Mater. Interfaces* **10,** 18012−18020 (2018).

[8] Ogi, S., Sugiyasu, K., Manna, S., Samitsu, S. & Takeuchi, M. Living supramolecular polymerization realized through a biomimetic approach. *Nat. Chem.* **6,** 188−195 (2014).

[9] Colomb-Delsuc, M., Mattia, E., Sadownik, J. W. & Otto, S. Exponential self-replication enabled through a fibre elongation/breakage mechanism. *Nat. Commnu.* **6,** 7427 (2015).

[10] Fukui, T. *et al*. Control over differentiation of a metastable supramolecular assembly in one

and two dimensions. *Nat. Chem.* **9,** 493−499 (2017).

[11] Ma, X. *et al*. Fabrication of chiral-selective nanotubular heterojunctions through living supramolecular polymerization. *Angew. Chem. Int. Ed.* **55,** 9539−9543 (2016).

[12] Kang, J. *et al*. A rational strategy for the realization of chain-growth supramolecular polymerization. *Science* **347,** 646−651 (2015).

[13] Zhanga, K., Yeunga, M. C. L., Leunga, S. Y. L. & Yam, V. W. W. Living supramolecular polymerization achieved by collaborative assembly of platinum(II) complexes and block copolymers. *PNAS* **114,** 11844−11849 (2017).

[14] Fukui, T., Sasaki, N., Takeuchi, M. & Sugiyasu, K. Living supramolecular polymerization based on reversible deactivation of a monomer by using a 'dummy' monomer. *Chem. Sci.* **10,** 6770−6776 (2019).

[15] Du, G., Moulin, E., Jouault, N., Buhler, E. & Giuseppone, N. Muscle-like supramolecular polymers: integrated motion from thousands of molecular machines. *Angew. Chem. Int. Ed.* **51,** 12504−12508 (2012).

[16] van der Schoot, P., Michels, M. A. J., Brunsveld, L., Sijbesma, R. P. & Ramzi, A. Helical Transition and Growth of Supramolecular Assemblies of Chiral Discotic Molecules. *Langmuir* **16,** 10076−10083 (2000).
