# Supplementary material for: Oriented arrangement of simple monomers enabled by confinement: towards living supramolecular polymerization
Source: Nat Commun. 2021 May 10;12:2596. doi: 10.1038/s41467-021-22827-4 (PMC8110532; doi:10.1038/s41467-021-22827-4)
Supplement: Supplementary file 3 — Description of Additional Supplementary Files [file 41467_2021_22827_MOESM3_ESM.pdf]

Description of Additional Supplementary Files

File Name: Supplementary Movie 1
Description: The vigorous move of metastable LSP_{20} without ultrasound kept for 0 min in FITS.
